# Iron derived from NCOA4-mediated ferritinophagy causes cellular senescence via the cGAS-STING pathway

**DOI:** 10.1038/s41420-023-01712-7

**Published:** 2023-11-18

**Authors:** Hong-Ying Li, Ting-Ting Wei, Miao Zhuang, Cheng-Ye Tan, Tian-Hua Xie, Jiping Cai, Yong Yao, Lingpeng Zhu

**Affiliations:** 1 Department of Ophthalmology, The Affiliated Wuxi People’s Hospital of Nanjing Medical University, Wuxi People’s Hospital, Wuxi Medical Center, Nanjing Medical University, Wuxi, China; 2 Center of Clinical Research, The Affiliated Wuxi People’s Hospital of Nanjing Medical University, Wuxi People’s Hospital, Wuxi Medical Center, Nanjing Medical University, Wuxi, China

**Keywords:** Senescence, Autophagy

## Abstract

Cellular senescence is a hallmark of aging and has been linked to age-related diseases. Age-related macular degeneration (AMD), the most common aging-related retinal disease, is prospectively associated with retinal pigment epithelial (RPE) senescence. However, the mechanism of RPE cell senescence remains unknown. In this study, tert-butyl hydroperoxide (TBH)-induced ARPE-19 cells and D-galactose-treated C57 mice were used to examine the cause of elevated iron in RPE cell senescence. Ferric ammonium citrate (FAC)-treated ARPE-19 cells and C57 mice were used to elucidated the mechanism of iron overload-induced RPE cell senescence. Molecular biology techniques for the assessment of iron metabolism, cellular senescence, autophagy, and mitochondrial function in vivo and in vitro. We found that iron level was increased during the senescence process. Ferritin, a major iron storage protein, is negatively correlated with intracellular iron levels and cell senescence. NCOA4, a cargo receptor for ferritinophagy, mediates degradation of ferritin and contributes to iron accumulation. Besides, we found that iron overload leads to mitochondrial dysfunction. As a result, mitochondrial DNA (mtDNA) is released from damaged mitochondria to cytoplasm. Cytoplasm mtDNA activates the cGAS-STING pathway and promotes inflammatory senescence-associated secretory phenotype (SASP) and cell senescence. Meanwhile, iron chelator Deferoxamine (DFO) significantly rescues RPE senescence and retinopathy induced by FAC or D-gal in mice. Taken together, these findings imply that iron derived from NCOA4-mediated ferritinophagy causes cellular senescence via the cGAS-STING pathway. Inhibiting iron accumulation may represent a promising therapeutic approach for age-related diseases such as AMD.

## Introduction

Age-related macular degeneration (AMD) is a serious vision-threatening eye disease predominantly accounting for blindness among people over 55 in developed countries [1]. AMD is divided into two types, neovascular (wet or exudative) and non-neovascular (dry or non-exudative). Dry AMD occurs in 80 ~ 90% of the cases and can cause permanent vision loss if left untreated [2]. Currently, clinical therapy for AMD is focused on alleviating choroidal neovascularization, which can improve vision in patients with wet AMD to a certain extent [3]. However, there is no treatment available for dry AMD patients. It is essential to investigate the underlying mechanism and identify potential targets for dry AMD.

Aging is the strongest individual factor for AMD risk. Retinal pigment epithelium (RPE) geographic atrophy and drusen deposition are clinical manifestations of dry AMD. Current evidences suggest that RPE dysfunction accounts for the development of dry AMD. Many senescent cells have been observed in the RPE layer of the elderly, suggesting that RPE cell senescence is closely associated with AMD [4]. Cellular senescence is a natural biological phenomenon, exhibiting permanent proliferation arrest in response to internal/external stresses. Senescence is regulated by triggers such as DNA damage, telomere disorders, oncogene activation and organelle stress [5]. The characteristic morphological and biochemical features of senescent cells include senescence-associated beta-galactosidase (SA-β-gal) activation, lipofuscin accumulation and senescence-associated secretory phenotype (SASP) [6]. Mitochondrial dysfunction reportedly plays a key role in cellular senescence [7]. The accumulation of damaged mitochondria in senescent cells is accompanied by a reduction in the efficiency of oxidative phosphorylation and an increase in reactive oxygen species (ROS) production. As a result, mitochondrial membrane permeability increases and mitochondrial DNA (mtDNA) is released into the cytosol. mtDNA has emerged as a significant trigger for the cyclic GMP-AMP synthase (cGAS)-cGAS-stimulator of interferon genes (STING) pathway [8]. The cGAS-STING pathway is well-recognized to be important for regulating senescence and associated SASP [9], although it is a well-established feature of aging cells, it remains unclear whether mitochondrial dysfunction is a cause or consequence of senescence.

Mitochondria are widely acknowledged as the key sites of cellular iron utilization [10]. Doxorubicin has been reported to increase mitochondrial iron accumulation, further increasing ROS production in the mitochondria [11]. A 20-year study found that levels of manganese, zinc and selenium are decreased, and iron, copper and iodine are increased in the aging population (aged 58–78 years) [12]. However, the physiological significance of this change has not been elucidated. Iron accumulation in the brain has been demonstrated in several neurodegenerative diseases, including Alzheimer’s and Parkinson’s [13]. Other studies found that iron could accumulate in the macula of AMD patients, particularly in RPE and Bruch’s membrane [14]. Besides, iron levels in the blood have been reported to be marginally higher in the AMD group [15]. Our previous study found that ARPE-19 cell death was accompanied by increased intracellular iron, ROS and lipid peroxidation levels [16]. The above evidence substantiates that iron may play an important role in RPE senescence.

Iron homeostasis is regulated by several pathways [17]: Fe^2+^ is oxidized to Fe^3+^, which can be bound to transferrin (Tf) to form the TF-TFR complex. The formation of this complex enables intracellular transportation. After entry into cells, Fe^3+^ can be dissociated from the Tf-TfR complex, thereby enabling Fe^3+^ to be reduced to Fe^2+^. Fe^2+^-free state is stored in ferritin heavy chain (FTH) in the cytoplasm or mitochondrial ferritin (FtMt) in the mitochondria. Fe^2+^ that is not utilized is then exported by ferroportin (Fpn/SLC40A1). Iron is recycled from ferritin by a process known as ferritinophagy [18]. As a specialized cargo receptor, nuclear receptor coactivator 4 (NCOA4) directly recognizes and binds to FTH1, then delivers iron-bound ferritin to autophagosomes for degradation. In light of NCOA4’s established role in intracellular iron homeostasis, there has been considerable interest in NCOA4’s role in the pathophysiological process associated with iron.

In this study, we systematically elucidated the cause of iron overload and the role of iron in aging in tert-butyl hydroperoxide (TBH)/ferric ammonium citrate (FAC)-treated ARPE-19 cells and D-galactose/FAC-treated C57 mice. Overall, our results suggest that iron plays an important role in RPE senescence. The findings imply that inhibiting iron accumulation might be a therapeutic target for age-related disease such as AMD.

## Results

### Iron is elevated during aging

**Fig. 1:**
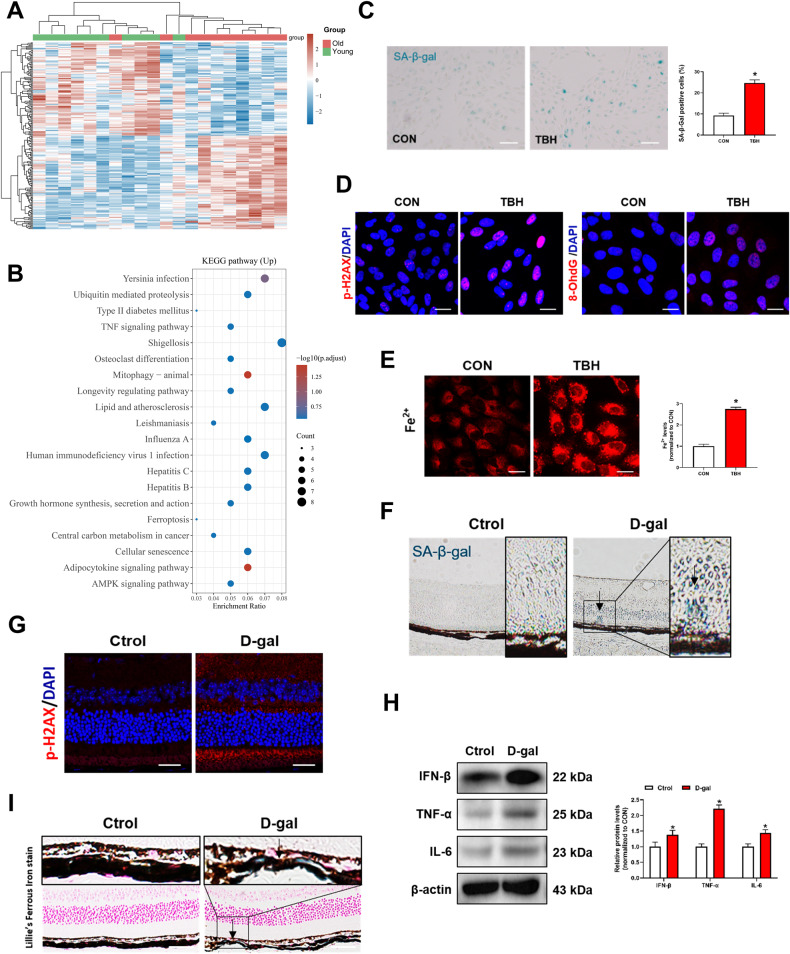
Iron is elevated during aging. **A** Heat map displays the changes of differential gene expression in two groups. Data were derived from Gene Expression Omnibus (GEO) data sets GSE58137. **B** KEGG pathway analysis of upregulated genes. ARPE-19 cells were treated with TBH (100 μM) for 48 h to induce cell senescence models. **C** Images of SA-β-Gal staining of ARPE-19 cells (*n* = 3). Scale bars, 100 μm. **D** Immunofluorescence staining of p-H2AX and 8-OhdG. Scale bars, 25 μm. **E** Intracellular Fe^2+^ levels were detected by the FerroOrange probe (1 μM, 30 min) (*n* = 3). Scale bars, 25 μm. **F** SA-β-Gal staining of mouse retina. Scale bars, 100 μm. **G** Immunofluorescence staining of p-H2AX in mouse retina. Scale bars, 25 μm. **H** The protein content of IFN-β, TNF-α and IL-6 in mouse retina was assessed by Western blot (*n* = 3). Mean ± SD. **I** lillie’s Ferrous Iron stain showing Fe^2+^ deposition in mouse retina. Blue depositions represent the accumulation of Fe^2+^. Scale bars, 100 μm. Data are shown as Mean ± SD. *p < 0.05 vs. control group.

To dissect the transcriptome landscape of the aging process, we downloaded the {type:entrez-geo, attrs:{text:GSE58137,term_id:58137}} GSE58137 dataset from the NCBI GEO DataSets. Based on age, 20 sex-matched participants were randomly assigned into two groups: Young Group (median age, 21 years) and Old Group (median age, 59 years). The basic information and the accession numbers of each patient are provided in Supplementary Table 4. Boxplot depicting the read counts normalization (Fig. S1A) and the distributions of age in two groups were presented in Fig. S1B. Then, we performed differential gene expression analysis in two groups and these genes were analyzed using the KEGG pathway database (http://www.genome.jp/kegg/) (Fig. 1A, B). We found that the KEGG ferroptosis signaling pathway is enriched. Ferroptosis is a kind of cellular death on the basis of iron accumulation. We wondered whether iron levels were altered during senescence. TBH is a stable and long-acting form of hydrogen peroxide. Hence, we established a senescent cell model of ARPE-19 using a low dose (100 μM) of TBH in vitro. As shown in Fig. 1C, the number of SA-β-gal positive cells (blue color) of the TBH-treated group was significantly higher than the control group. Oxidative DNA damage during aging was assessed by immunofluorescence of phosphorylated histone H2AX (p-H2AX) and 8-hydroxy-2’-deoxyguanosine (8-OhdG) [19]. The results showed elevated expressions of p-H2AX and 8-OhdG in TBH-treated ARPE-19 cells (Fig. 1D). Notably, intracellular iron levels were significantly increased in TBH-treated ARPE-19 cells (Fig. 1E), which suggests that iron elevation may be related to ARPE-19 cell senescence. Next, we used D-galactose (D-gal) to induce senescence in vivo. SA-β-gal-positive cells were significantly elevated in the D-gal-treated mice (Fig. 1F). Amyloid β (Aβ) and p-tau are acknowledged as senescence biomarkers [20]. Our study also found elevated Aβ and p-tau in retinal tissues and brain tissues of D-gal-treated mice (Fig. S2A, B), which further indicating the success of aging model establishment. Immunofluorescence showed increased p-H2AX expression in the retina of the D-gal-treated group (Fig. 1G). As shown in Fig. 1H, expressions of senescence-related proteins (IFN-β, TNF-α, IL-6) were elevated in the D-gal-treated mice. Interestingly, we found iron deposition in the retina of the D-gal group (Fig. 1I). These results corroborate that iron level increase during the senescence process.

### NCOA4 mediates the elevation of iron in senescent ARPE-19 cells

**Fig. 2:**
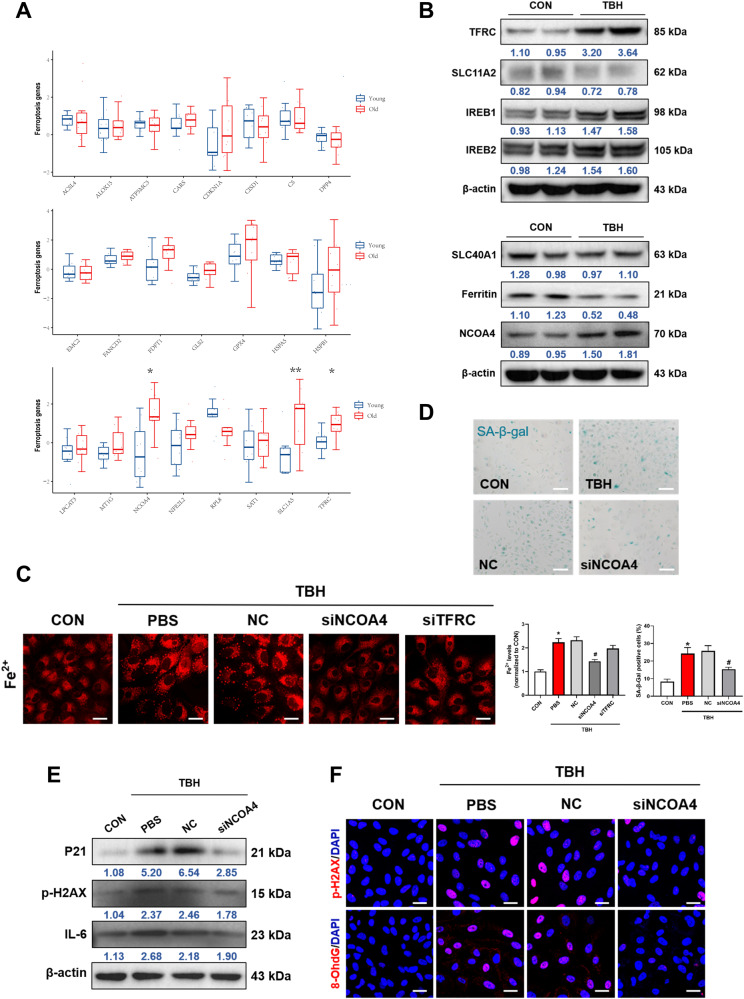
NCOA4 mediates the elevation of iron in senescent ARPE-19 cells. **A** The analysis of expression levels of 24 ferroptosis-associated genes from GEO datasets (GEO58137). **B** The expression of iron metabolism-related proteins was detected by Western blot (*n* = 3). **C** Intracellular Fe^2+^ was detected after the knockdown of NCOA4 by the FerroOrange probe (1 μM, 30 min) (*n* = 3). Scale bars, 25 μm. **D** The expression of SA-β-Gal staining positive cells were ameliorated after knockdown of NCOA4 (*n* = 3). Scale bars, 100 μm. **E** The expression of P21, p-H2AX and IL-6 were ameliorated after knockdown of NCOA4 (*n* = 3). **F** Immunofluorescence staining of p-H2AX and 8-OhdG. Scale bars, 25 μm. Data are shown as Mean ± SD. *p < 0.05 vs. control group. ^#^*p* < 0.05 vs. TBH group.

Further, we sought to investigate the molecular mechanism governing the aberrant elevation of iron in aging. The expression levels of 24 ferroptosis-associated genes including ACSL4, ALOX15, CARS, ATP5MC3, CDKN1A, CISD1, CS, DPP4, EMC2, FANCD2, FDFT1, GLS2, GPX4, HSPA5, HSPB1, LPCAT3, MT1G, NCOA4, NFE2L2, RPL8, SAT1, SLC1A5, SLC7A11 and TFRC from GEO datasets (GEO58137) were analyzed. We found that NCOA4, SLC1A5 and TFRC expression levels were significantly increased in the elderly group (Fig. 2A). Next, we examined the expressions of iron homeostasis-related proteins in senescent ARPE-19 cells. We found that the level of TFRC, an indicator of iron uptake, was elevated and ferritin, the major iron-storage protein, was decreased in TBH-treated cells. Notably, NCOA4, a selective autophagy receptor for ferritin, was also increased in TBH-treated cells. However, no significant differences were observed in other iron homeostasis-related proteins (Fig. 2B). To further evaluate the role of TFRC and NCOA4 in the elevation of iron, specific siRNAs were used. Knockdown efficiency was proven by western blot (Fig. S3A). As shown in our results, iron content was reduced in the NCOA4 knockdown group, but no significant difference was found in the TFRC knockdown cells (Fig. 2C). Besides, the number of SA-β-gal-positive cells was significantly reduced in the NCOA4 knockdown group (Fig. 2D). Besides, our results indicated that knockdown of NCOA4 reverses the upregulation of senescence biomarkers such as P21, p-H2AX and IL-6 under senescent condition (Fig. 2E). Immunofluorescence was performed to confirm the expressions of p-H2AX and 8-OhdG and we found that NCOA4 knockdown rescued DNA damage (Fig. 2F). The above results suggest that NCOA4 is involved in the elevation of iron in senescent ARPE-19 cells.

### NCOA4 mediates iron elevation via autophagic degradation of ferritin

**Fig. 3:**
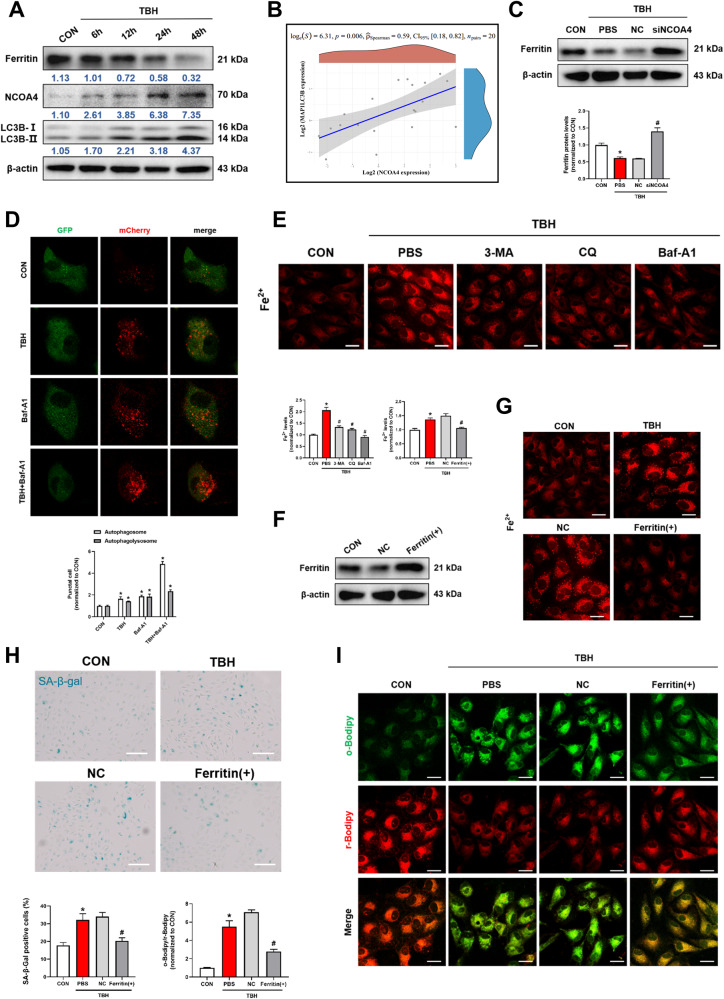
NCOA4 mediates iron elevation via autophagic degradation of ferritin. **A** The Ferritin, NCOA4 and LC3B were evaluated by western blot. ARPE-19 cells were treated with TBH (100 μM) for different time durations (6, 12, 24, and 48 h) (*n* = 3). **B** NCOA4 expression was positively correlated with LC3B in human blood samples. (GEO accession numbers: GSE58137). **C** The protein expression of ferritin was markedly increased after knockdown of NCOA4 (*n* = 3). **D** Live-cell imaging of ARPE-19 cells expressing Ad-mCherry-GFP-LC3B. Cells were incubated in control or presence of TBH (100 μM)/Bafilomycin A1 (Baf-A1) (100 nM)/TBH + Baf-A1 for 48 h prior to imaging (*n* = 3). Scale bars, 25 μm. **E** Autophagy inhibitors, including 3-Methyladenine (3-MA, 5 mM), Chloroquine (CQ, 5 μM) and Baf-A1 (100 nM), reversed the TBH-induced increases intracellular Fe^2+^ levels (*n* = 3). Scale bars, 25 μm. **F** Ferritin was overexpressed by FTH1 cDNA transfection and overexpression efficiency was detected by Western blot. **G** Overexpression of ferritin reduced the intracellular Fe^2+^ levels (*n* = 3). Scale bars, 25 μm. **H** SA-β-Gal staining positive cells were decreased after ferritin overexpression (*n* = 3). Scale bars, 100 μm. **I** The intracellular lipid peroxidation levels were detected by C11-BODIPY probes (10 μM, 30 min), reduced (red) and oxidized (green) Bodipy (*n* = 3). Scale bars, 25 μm. Data are shown as Mean ± SD. *p < 0.05 vs. control group. ^#^*p* < 0.05 vs. TBH group.

Ferritin is well-recognized as the major iron storage protein of vertebrates [21]. During ferritinophagy, NCOA4 directly binds and delivers iron-bound ferritin to autophagosomes for degradation and causes the release of iron from ferritin [22]. In the present study, the level of NCOA4 was elevated and ferritin was reduced time-dependently in TBH-treated ARPE-19 cells, we also found LC3B, an important autophagy-related protein, was upregulated in senescent cells (Fig. 3A). Furthermore, we confirmed that NCOA4 expression was positively correlated with LC3B in the collected 20 human blood samples (Fig. 3B). Knockdown of NCOA4 significantly increased the expression of ferritin, further clarifying that NCOA4-mediated the degradation of ferritin (Fig. 3C). Autophagy is a dynamic process and integrity of autophagy flow maintaining the cell physiological functioning. Next, mCherry-GFP-LC3B adenovirus was used to monitor autophagic activity and the progressive appearance of red puncta indicates the activation of autophagy [23]. Bafilomycin A1 (Baf-A1), a vacuolar ATPase inhibitor, increased the number of puncta containing mCherry and GFP, while TBH further increased autophagic flux (Fig. 3D). Notably, a significant down-regulation of intracellular iron was observed after treatment with autophagy inhibitors, including 3-Methyladenine (3-MA) Chloroquine (CQ) and Baf-A1 (Fig. 3E). In addition, autophagy inhibitors also downregulated intracellular lipid peroxidation levels (Fig. S4A). The above results showed that activation of autophagy plays a central role in iron aggregation.

To further define the biological effect of ferritin, ARPE-19 cells were transfected with FTH1 cDNA (Fig. 3F). Overexpression of ferritin significantly reduced the content of iron in TBH-treated ARPE-19 cells (Fig. 3G), further confirming that ferritin is negatively correlated with intracellular iron levels. Concurrently, ferritin overexpression reduced the number of SA-β-gal-positive cells (Fig. 3H). C11-BODIPY staining showed that intracellular lipid peroxidation was increased in senescent cells, while ferritin overexpression significantly inhibited the elevation (Fig. 3I). In conclusion, our study revealed that NCOA4 mediates iron elevation through autophagic degradation of ferritin in senescent state in vitro.

### Iron causes mitochondrial dysfunction and accelerates ARPE-19 cell senescence via the cGAS-STING signaling pathway

**Fig. 4:**
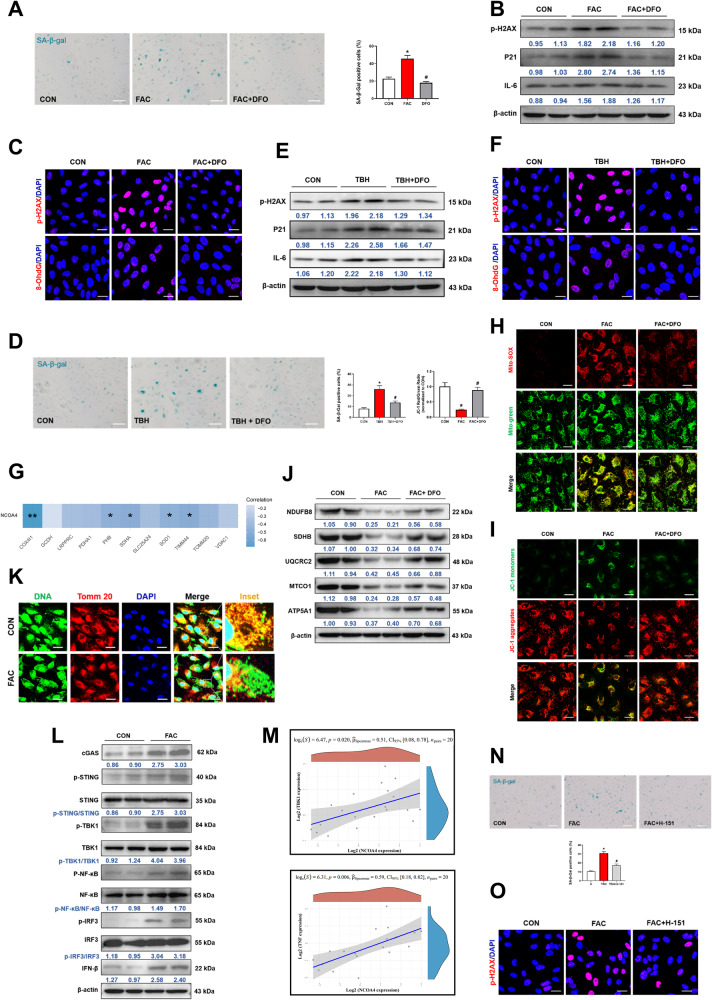
Iron causes mitochondrial dysfunction and accelerates ARPE-19 cell senescence via the cGAS-STING signaling pathway. ARPE-19 cells were pretreated with deferoxamine (DFO, 20 μM) for 2 h, followed by stimulation with TBH (100 μM) or ferric ammonium citrate (FAC, 200 μM) for 48 h. **A** DFO treatment reduced the number of SA-β-Gal positive cells in FAC group (*n* = 3). Scale bars, 100 μm. **B** DFO reduced the expression levels of aging-related proteins (p-H2AX, P21 and IL-6) in FAC group by Western blot (*n* = 3). **C** Immunofluorescence of p-H2AX and 8-OhdG in FAC group. Scale bars, 25 μm. **D** DFO treatment reduced the number of SA-β-Gal staining positive cells in TBH group (*n* = 3). Scale bars, 100 μm. **E** DFO reduced the expression levels of aging-related proteins (p-H2AX, P21 and IL-6) in TBH group by Western blot (*n* = 3). **F** Immunofluorescence of p-H2AX and 8-OhdG in TBH group. Scale bars, 25 μm. **G** Mitochondrial functions-associated genes including COX4I1, PHB, SDHA, SOD1 and TIMM44 was negatively correlated with NCOA4 in human blood samples, respectively. (GEO accession numbers: GSE58137). **H** MitoSOX™ Red (5 μM, 30 min) and Mito-Tracker Green (200 nM, 30 min) were used to co-localize mitochondrial ROS and mitochondria, respectively. Scale bars, 25 μm. **I** Mitochondrial membrane potential was assessed by JC-1 staining. Scale bars, 25 μm. **J** OXPHOS activity was detected by Western blot (*n* = 3). **K** Immunofluorescence of dsDNA and TOMM20 colocalization. Scale bar, 25 μm. **L** Detection of cGAS-STING pathway-related proteins by Western blot (*n* = 3). **M** The expression levels of TBK1 and TNF both had a positive significant correlation with NCOA4 in the collected 20 human blood samples. ARPE-19 cells were pretreated with STING inhibitor H-151 (10 μM) for 2 h, followed by stimulation with FAC (200 μM) for 48 h. **N** H-151 reversed FAC-induced increase in SA-β-Gal staining positive cells (*n* = 3). Scale bars, 100 μm. **O** H-151 rescued FAC-induced up-regulation of p-H2AX in cell by immunofluorescence. Data are shown as Mean ± SD. *p < 0.05 vs. control group. ^#^*p* < 0.05 vs. TBH or FAC group.

The above results revealed the cause of elevated iron in senescent ARPE-19 cells. Next, we investigated the relevance of iron overload in cells. Interestingly, we found that FAC-treated cells tended to have a SA-β-gal-positive phenotype (Fig. 4A) and aging-related proteins such as p-H2AX, P21, IL-6 and 8-OhdG increasement (Fig. 4B, C). DFO, an iron chelator, reversed FAC-induced senescence-associated phenotypes (Fig. 4A–C). Besides, DFO also alleviated FAC-induced lipid peroxidation (Fig. S4B). The above results implied that iron overload may play a key role in RPE cell senescence. To further investigate the role of iron in senescence, DFO was also used in TBH-induced aging cells. DFO significantly reduced the number of SA-β-gal-positive cells and decreased the expressions of p-H2AX, P21, IL-6 and 8-OhdG (Fig. 4D–F).

Next, we sought to ascertain the molecular mechanisms underlying iron-induced senescence. Mitochondrial dysfunction is closely linked to aging-related processes [24]. Furthermore, we found that 5 mitochondrial functions-associated genes including COX4I1, PHB, SDHA, SOD1 and TIMM44 were negatively correlated with NCOA4, respectively, in the collected 20 human blood samples (Figs. 4G and S5A), indicating that impaired mitochondrial function is closely related to iron accumulation in aging. ROS levels were elevated in the mitochondria after FAC treatment (Fig. 4H). Moreover, FAC decreased the mitochondrial membrane potential (Fig. 4I). A significant decrease in mitochondrial OXPHOS complexes in FAC-treated cells was observed (Fig. 4J). DFO treatment could reverse mitochondrial dysfunction caused by FAC (Fig. 4H–J). We also examined the level of ferritinophagy and found that Mito-T treatment decreased NCOA4 expression and accompanied by an increase in ferritin (Fig. S6A). In summary, our results suggested that autophagic degradation of ferritin is associated with mitochondrial dysfunction. Collectively, our results showed iron overload-induced senescence of ARPE-19 cells is associated with mitochondrial dysfunction.

As indicated above, iron accumulation, mitochondrial dysfunction and senescence-associated inflammation secretory phenotypes are closely related. Previous research has shown that cGAS-STING signaling plays a crucial role in mitochondrial dysfunction and inflammatory response, as well as in aging. cGAS-STING signaling activates transcription factors such as NF-κB and IRF3, and promotes inflammatory responses. NF-κB regulates the expressions of TNF-α and IL-6, and IRF3 regulates IFN-β. These are three main inflammatory factors in aging. Therefore, we speculated that iron-induced cellular senescence may be related to the cGAS-STING signaling pathway. The immunofluorescence assay showed that mtDNA is released from damaged mitochondria to cytoplasm (Fig. 4K). It has been shown that mtDNA in the cytoplasm triggers SASP by activating the cGAS-STING pathway [25]. Next, we evaluated the expressions of cGAS-STING pathway-related proteins. As shown in Fig. 4L, cGAS and phosphorylated STING were upregulated by FAC. This elevation is consistent with the upregulation of phosphorylated TBK1, phosphorylated NF-κB, phosphorylated IRF3 and IFN-β, which are downstreams of STING. The above experiments suggested that FAC-induced senescence through cGAS-STING pathway. The expression levels of TBK1 and TNF both had a positive significant correlation with NCOA4 in the collected 20 human blood samples further confirmed the above results (Fig. 4M). H-151, a STING inhibitor, significantly reversed FAC-induced senescence phenotypes, including SA-β-gal-positive cells and p-H2AX expression (Fig. 4N, O). Thus, we concluded that iron leads to mitochondrial dysfunction, which causes cGAS-STING signaling pathway activation and consequent cell senescence.

### Retinal iron deposition causes senescence in C57 mice

**Fig. 5:**
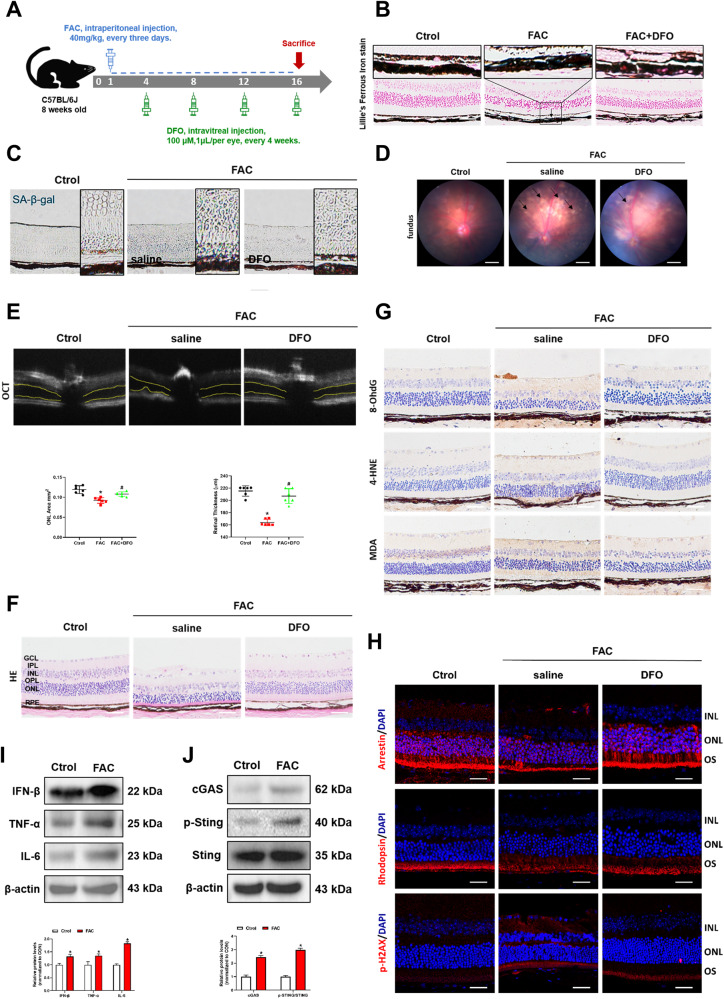
Retinal iron deposition causes senescence in C57 mice. **A** Experimental timeline of FAC and DFO treatment in C57 mice. **B** Lillie’s Ferrous Iron stain showing Fe^2+^ deposition in retinal tissue of mice. Scale bars, 100 μm. **C** SA-β-Gal staining of mouse retina. Scale bars, 100 μm. **D** The fundus photograph of mice. Scale bars, 500 μm. **E** The outer nuclear layer (ONL) area in horizontal optical coherence tomography scans (yellow line) was quantified by ImageJ (*n* = 3). **F** The H&E staining of mouse retina (*n* = 3). Scale bars, 100 μm. **G** The immunohistochemistry analysis of 8-OhdG, 4-HNE and MDA in the mouse retina. Scale bars, 100 μm. **H** The protein expression of Arrestin, Rhodopsin and p-H2AX in mouse retina was evaluated by immunofluorescence. Scale bars, 25 μm. **I** The protein content of IFN-β, TNF-α and IL-6 in eye tissue of mice was assessed by Western blot (*n* = 3). **J** The protein content of cGAS, p-STING and STING in eye tissue of mice was assessed by Western blot (*n* = 3). Data are shown as Mean ± SD. *p < 0.05 vs. control group. ^#^*p* < 0.05 vs. FAC group.

Experiments above confirmed that iron can induce cell senescence in vitro. In order to further investigate the effect of iron in mouse retinas, we established an iron overload model in C57BL/6J mice (Fig. 5A). Lillie’s Ferrous Iron stain demonstrated that intraperitoneal injection of FAC resulted in retinal iron deposition, while DFO reversed retinal iron accumulation (Fig. 5B). The number of SA-β-gal-positive cells was significantly increased in the FAC group, while DFO treatment reversed this phenomenon (Fig. 5C). Aβ and p-tau are senescence biomarkers as described previously. We found elevated Aβ and p-tau in retinal and brain tissues in FAC-treated mice (Fig. S7A, B). The results suggested that iron is associated with aging in vivo. Senescent RPE cells leads to the accumulation of drusen, causing retinal dysfunction and promoting the development of AMD. Interestingly, Aβ and p-tau, are also found in drusen-like substance in RPE layer in AMD. Our results showed that DFO rescued FAC-induced Aβ and p-tau elevation in retinal (Fig. S7A, B), further confirming iron causes RPE senescence in AMD.

RPE senescence can lead to a multitude of physiological changes in retina. We found that the white drusen-like substances in the fundus were increased in the FAC-treated mice and they were significantly decreased after DFO treatment (Fig. 5D). The outer nuclear area (yellow lines) and retinal thickness were significantly decreased after FAC treatment, whereas DFO abolished the alteration (Fig. 5E, F). Elevated 4-HNE, 8-OhdG and MDA levels represent the occurrence of oxidative stress in the retina. As the major components of drusen, they are also upregulated during aging process. 4-HNE, 8-OhdG and MDA were elevated in FAC-treated mice, and DFO rescued this elevation (Fig. 5G). Declines in Rhodopsin (a compound of retinoids and optins) and Arrestin (Retinoic acid inhibitor protein) in the macula are manifestations of progressive AMD [26]. As shown in Fig. 5H, we found a decrease in Arrestin and Rhodopsin in FAC-treated mice, while their expressions were significantly upregulated by DFO. These results further confirmed that retinal iron deposition causes RPE cell senescence and AMD-like retinal alterations. p-H2AX is associated with oxidative DNA damage during aging. DFO downregulated p-H2AX expression in FAC-treated mouse retinas (Fig. 5H). The expressions of SASP, including IFN-β, TNF-α and IL-6, were also upregulated in FAC-treated mice (Fig. 5I). Mechanistically, FAC promotes SASP by activating the cGAS-STING signaling pathway (Fig. 5J), which are consistent with the in vitro results. Therefore, we conclude that retinal iron overload causes senescence and AMD-like retinal alterations in mice.

### DFO ameliorates D-gal induced senescence in C57 mice

**Fig. 6:**
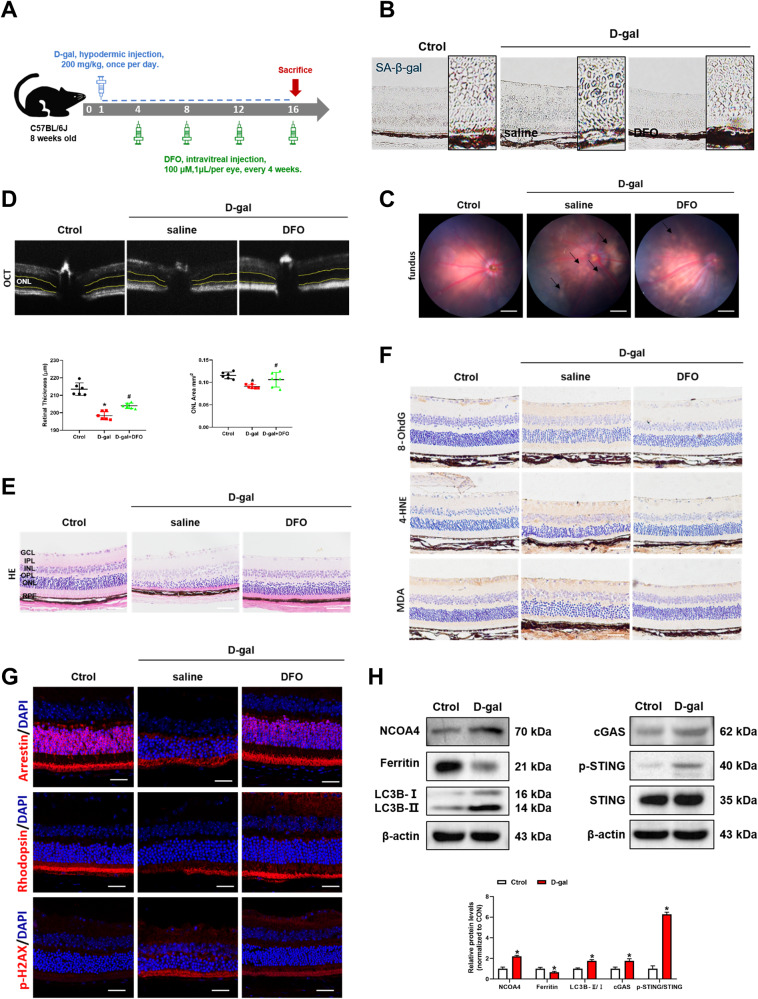
DFO ameliorates D-gal induced senescence in C57 mice. **A** Experimental timeline of D-gal and DFO treatment in C57 mice. **B** SA-β-Gal staining of mouse retina. Scale bars, 100 μm. **C** The fundus photograph of mice. Scale bars, 500 μm. **D** The outer nuclear layer (ONL) area in horizontal optical coherence tomography scans (yellow line) was quantified by ImageJ (*n* = 3). **E** The H&E staining of mouse retina (*n* = 3). Scale bars, 100 μm. **F** Immunohistochemistry analysis of 8-OhdG, 4-HNE and MDA in the mouse retina. Scale bars, 100 μm. **G** Arrestin, Rhodopsin and p-H2AX in the mouse retina was evaluated by immunofluorescence. Scale bars, 25 μm. **H** The protein content of NCOA4, Ferritin, LC3B, cGAS, p-STING and STING in the mouse retina was assessed by Western blot (*n* = 3). Data are shown as Mean ± SD. *p < 0.05 vs. control group. ^#^*p* < 0.05 vs. D-gal group.

To further investigate the role of iron in senescence, iron chelator was used in D-gal-induced aging mice (Fig. 6A). The number of SA-β-gal-positive cells was significantly reduced in the DFO-treated mice compared to the D-gal group (Fig. 6B). White drusen-like substances in the fundus were increased in D-gal-treated mice but were rescued by DFO administration (Fig. 6C). Besides, DFO treatment reversed D-gal-induced up-regulation of Aβ and p-tau in mouse retinas (Fig. S2A, B). The above results confirmed the enhanced role of iron in the cellular senescence.

The outer nuclear area and retinal thickness were significantly decreased in D-gal-treated mice according to OCT and HE staining, while DFO mitigated the retinal structure alterations (Fig. 6D, E). 8-OhdG, 4-HNE and MDA were elevated after D-gal treatment (Fig. 6F). Decreases in Arrestin, Rhodopsin and increases in p-H2AX were observed in the retinas of D-gal group (Fig. 6G). In contrast, the above phenomena were significantly improved after DFO treatment (Fig. 6F, G). The above experiments proved that iron causes retinal dysfunction and AMD-like retinal alterations in D-gal-induced aging mice.

In addition, we noticed that the increased levels of NCOA4 and LC3B were accompanied by degradation of ferritin in the D-gal-treated group. Besides, cGAS and phosphorylated STING were upregulated in D-gal-treated mice (Fig. 6H). These results further confirmed the role of NCOA4-mediated autophagic degradation of ferritin in senescent cells and supported that iron induces cellular senescence through cGAS-STING signaling pathway. In summary, our results suggested that iron plays an enhanced role in cellular senescence.

## Discussion

**Fig. 7:**
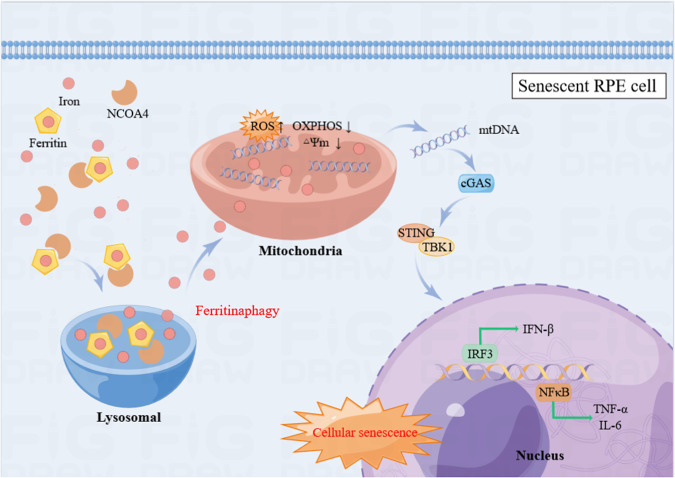
Iron derived from NCOA4-mediated ferritinophagy causes retinal pigment epithelium senescence. Our flow chart illustrates that iron elevation causes retinal pigment epithelium senescence. NCOA4-mediated autophagic degradation of ferritin and induced iron accumulation. Iron accumulation leading to mitochondrial dysfunction, and mtDNA is released from damaged mitochondria to cytoplasm. Cytoplasm mtDNA further activates the cGAS-STING pathway, which promotes SASP and induces cell senescence. The figure is drawn by Figuredraw.

Our study focused on the cause and relevance of iron increases in RPE cells. Firstly, we substantiated iron elevation in aging models, and NCOA4-mediated autophagic degradation of ferritin prompted iron release from ferritin. Secondly, our experimental results suggest that iron overload leads to mitochondrial dysfunction and upregulates the cGAS-STING pathway, and ultimately accelerates RPE cell senescence. In addition, we showed that FAC induced AMD-like changes in mice, and iron chelator reversed the senescence phenotypes and AMD-like retinal alterations in D-gal or FAC-treated mice. These results suggested that iron induced cellular senescence and promoted the progression of AMD. The main mechanisms are shown in Fig. 7.

Cellular senescence involves various biological processes, such as embryonic development, wound healing or repair, tumorigenesis, aging and age-related diseases [27]. Previous studies reported that protein levels of p21 and p53, two typical senescence indicators, were increased in RPE cells isolated from elderly donors (84–86 years old) [28]. Senescent cells have been detected in retinal and RPE cells of older adults. It has been shown that risk factors of AMD, including oxidative stress, DNA damage and mitochondrial ROS, are also associated with cellular senescence [29]. The relationship between senescence and AMD has attracted much interest in recent years. Given that RPE senescence is an early indicator of AMD, it is important to understand the underlying mechanisms that influence RPE senescence [30]. A study demonstrated that AMD patients have more iron and iron carrier transferrin in the retina, indicating that retinal iron homeostasis may be critical in the development of AMD [31]. However, the mechanisms underlying iron accumulation and its biological effects on the retina remain poorly understood.

Under physiological conditions, moderate levels of ROS have been associated with cellular senescence [32]. TBH is an inducer of oxidative stress, which leads to the slow release of peroxides. Ample evidence suggests that TBH can induce senescence in myeloid cells and human skin fibroblasts [33, 34]. It has also been reported that phenotypic features of AMD can be observed in TBH or H_2_O_2_ treated-ARPE-19 cells [35]. Therefore, we established an ARPE-19 cell senescence model with TBH. At present, many indicators are available for the detection of senescence. SA-β-gal staining is a key indicator for detecting cellular senescence. P21 as a marker of cell cycle arrest, p-H2AX and 8-OhdG as symbols of oxidative DNA damage, and IL-6, TNF-α, IFN-β as components of SASP, which are typical indicators of senescence [36]. 4-HNE and MDA are lipid peroxidation products that usually accumulate in senescent cells [37]. We also found their accumulation in TBH-treated cells (Fig. S8A).

Iron deposition was found in senescent cells. Iron accumulation plays an important role in many retinal diseases as well as cellular senescence. As a potent generator of hydroxyl radicals, iron is critical for many metabolic processes. The expression of transferrin, transferrin receptor, ferritin, and iron regulatory proteins has been demonstrated in the human retina. The level of iron in the retinal macula increases with age and contributes to the potential for retinal degeneration [38]. Iron accumulation leads to oxidative stress and cell death, which are associated with aging and neurodegenerative diseases. Current evidence suggests that many receptors and proteins regulate iron homeostasis in cells. Over 60% of total iron is present as heme iron, and different proteins regulate iron uptake, storage, and export. Cellular uptake of iron occurs in the form of free iron bound to the transferrin receptor (TfR) [39]. Our study found that TfR was upregulated in aging ARPE-19 cells. However, no significant reduction of intracellular iron was observed after the knockdown of TfR. We hypothesize that cells may mediate iron uptake by regulating the expression of TfR on the cell membrane, but the overall intracellular iron level is not affected due to the compensatory mechanisms. SLC11A2 is a divalent metal transporter located on the endosomal membrane, which can transport iron from the endosomal membrane to the cytoplasm [40]. Moreover, unused or non-stored iron in cells is excreted through SLC40A1, the cell membrane ferroportin protein [41]. In senescent ARPE-19 cells, SLC11A2 and SLC40A1 were slightly downregulated, indicating that iron excretion was reduced to some extent. Moreover, iron regulatory proteins, IREB1 and IREB2, can regulate cellular iron concentration by regulating the mRNA level of ferritin [42]. Another study documented that their expressions were positively correlated with TFRC gene expression and negatively correlated with the mRNA level of SLC40A1 [43]. Ferritin is the major intracellular iron storage protein complex, including FTL1 (ferritin light polypeptide 1) and FTH1 (ferritin heavy polypeptide 1) and elevated expression of ferritin limits iron deposition [44]. Our study shows ferritin expression is negatively correlated with intracellular iron levels and cell senescence. NCOA4 is a critical cargo receptor for the autophagic degradation of ferritin, which causes iron release, a process known as ferritinophagy [45]. Consistent with the literature [46], we found a time-dependent increase in NCOA4 accompanied by ferritin degradation in aging ARPE-19 cells. Importantly, knockdown of NCOA4 significantly decreased the intracellular iron levels and cell senescence. The accumulation of 4-HNE and MDA was ameliorated after the knockdown of NCOA4 and ferritin overexpression (Fig. S8B). The above findings indicate that NCOA4 and ferritin have significant regulatory effects on iron homeostasis. The specific mechanism remains unclear and warranting further investigation.

Autophagy is an evolutionarily conserved degradation pathway whereby lysosomes degrade components, including nucleic acids, proteins, lipids and organelles, for maintaining homeostasis [47]. In the present study, we documented the upregulation of autophagy-related protein LC3B as well as autophagic flux in aging ARPE-19 cells. A significant down-regulation of intracellular iron was observed after treatment with autophagy inhibitors. The above results imply that the activation of the autophagy plays a central role in iron aggregation.

Mitochondria are involved in the progression of cellular senescence [48]. Mitochondrial dysfunction and iron accumulation are typical features of the senescence process. Mitochondrial dysfunction is defined as reduced respiratory chain function and reduced mitochondrial membrane potential, usually accompanied by increased oxygen radical production [49]. Both iron accumulation and mitochondrial dysfunction can form a malignant cycle in which the accumulation of non-heme iron during aging disrupts cellular homeostasis and leads to the progression of various neurodegenerative diseases [50]. Oxidative stress-induced RPE damage is the basis of AMD progression. RPE cells are constantly exposed to oxidative stress, which may lead to the accumulation of damaged cellular proteins, lipids, nucleic acids and organelles [51]. Damaged mitochondrial biodegradation results in mitochondrial dysfunction, energy deficiency and elevated susceptibility to death. Our experiments demonstrate that iron accumulation, mitochondrial dysfunction and SASP are intimately linked. The mitochondrial permeability transition pore (mPTP) is more susceptible to opening and release of mtDNA into the cytoplasm under aging. cGAS-STING signaling is a key cytoplasmic DNA sensor in innate immunity [52]. Although cGAS-STING signaling normally mediates immune surveillance and is neuroprotective, excessive activation of it may be detrimental and is closely associated with aging and age-related neurological disorders. cGAS-STING plays an important role in both mitochondrial dysfunction and inflammatory response as well as in aging. It has been revealed that the cytoplasmic mtDNA in senescent cells triggers SASP by activating the cGAS-STING pathway. cGAS-STING signaling pathway further activates the transcription factors NF-κB and IRF3 and promotes the expressions of SASP. Cytoplasmic mtDNA promotes inflammatory responses in senescent cells, and the removal of cytoplasmic DNA decreases the activity of SA-β-gal [53]. Interestingly, iron is a major component of Fe-S clusters or heme synthesis essential for maintaining the mitochondrial respiratory chain or tricarboxylic acid cycle [54]. Moreover, it has been shown that excess iron can damage mitochondria and mtDNA in rats [55]. Our research concluded that iron leads to mitochondrial dysfunction and subsequent activation of cGAS-STING signaling pathway, ultimately causing cell senescence. Interestingly, we also found that mitochondrial iron is significantly elevated in senescent RPE cells (Fig. S9A), but the sources of iron in mitochondria remain poorly defined. However, it is clear that elevated mitochondrial iron can further induce mitochondrial dysfunction. Intracellular iron can be transported from cytoplasm to mitochondria via the mitochondrial membrane proteins such as SLC25A28 and SLC25A37. Whether iron can be transported in mitochondria in other ways is still unknown at present and warrants further investigation in the future. mPTP is a group of protein complexes that exist in the mitochondrial membranes. We suggest that the opening of mPTP may also be one of the reasons for the entry of iron into mitochondria.

Earlier studies found that intraocular FAC injections induced oxidative stress and geographic atrophy-like retinal degeneration [56]. However, it should be noted that direct intraocular injection of FAC inevitably leads to retinal tissue damage. It is noteworthy that the iron level in the plasma increased slowly with age. Long-term intraperitoneal injection of FAC may exert a similar effect to iron deposition in the elderly. Therefore, we established an iron-overload mice model with a long-term intraperitoneal injection of FAC. This model successfully induced iron deposition in the retina and showed cellular senescence phenotypes and AMD-like retinal alterations. In the D-gal-induced aging mice, we also found increased deposition of iron in the retina and further investigated the role of iron in aging. Aβ and p-Tau, two aging-related proteins as well as components of drusen. Accumulation of Aβ and p-tau appear in the brain of Alzheimer’s and neuroinflammation condition [57]. Consistent with this, we also detected upregulation of Aβ and p-tau in the brain and retina of iron overload or D-gal-induced aging model in vivo. Besides, iron chelator rescued AMD-like retinal alterations and senescence phenotypes, which further confirming the important role of iron in aging. Notably, D-gal-induced aging in mice also shows AMD-like retinal alterations, perhaps it may serve as a novel model for AMD.

The RPE cells play a critical role in the progression of AMD. Senescent RPE cells have been detected in AMD and therapies targeting senescent cells are able to ameliorate retinal degeneration.

Iron accumulation and mitochondrial damage play essential roles in the progression of AMD in RPE cells. Iron exists as Fe^2+^, heme or iron-sulfur clusters and plays a key role at the center of glycolysis and the tricarboxylic acid cycle. As a transition metal, iron promotes free radical formation through the Fenton reaction, and iron-dependent lipid peroxidation drives the regulation of many pathophysiological processes [58]. Mitochondria are the center of oxygen utilization and energy production, which essentially leads to the production of ROS [59]. Mitochondrial aging associated with ROS has also been reported to be caused by the disruption of iron homeostasis [60]. Mitochondrial oxygen consumption in conjunction with the electron transport chain is a major function of mitochondrial energy production, in which iron plays an essential role [61]: (1) the electron transport chain produces ATP with the help of iron; (2) heme protein biosynthesis; (3) key cofactors required for iron-sulfur cluster formation. In addition, dysregulation of mitochondrial metal ions and ROS is thought to alter the cytoplasmic environment and mitochondrial membrane integrity, affecting with mitochondrial function and leading to mitochondrial dysfunction and energy depletion [62]. Increased iron in mitochondria with age, particularly under oxidative stress, may be a potential cause of age-related mitochondrial dysfunction. Our study reveals the vital role of iron in senescent RPE cells in vivo and in vitro. However, the present study has a few limitations. Firstly, we detected elevated mitochondrial iron in senescent RPE cells, however, it remains unclear whether iron elevation first occurred in the mitochondria or the cytoplasm. Secondly, how iron gain entry into the mitochondria remains unclear. Thirdly, an iron overload/chronic aging model of mice was used in our experiments. Although it may better resemble the pathology of AMD, disparities in the pathophysiology of AMD remain. Further investigations, including clinical samples, are warranted to confirm our results. However, in spite of these limitations, our results reveal the mechanism of elevated iron in aging RPE cells and found that iron deposition in the retina may be a critical contributor of AMD. This study provides compelling evidence of the value of iron as a potential therapeutic target for AMD.

## Materials and methods

### Materials and reagents

Tert-butyl hydroperoxide (TBH) were purchased from Sigma-Aldrich (St. Louis, USA). Ferric ammonium citrate (FAC) and D-galactose (D-gal) was obtained from Selleck (Shanghai, China) and Aladdin (Shanghai, China), respectively. Deferoxamine (DFO), Mito-TEMPO (Mito-T), 3-Methyladenine (3-MA), Chloroquine (CQ), Bafilomycin A1 (Baf-A1), and H-151 were purchased from MedChemExpress (NJ, USA). FerroOrange and MitoPeDPP were purchased from Dojindo (Japan). C11-BODIP (581/591) probes and MitoSOX™ Red was obtained from Thermo Fisher (USA). SA-β-Gal Staining Kit, AdPlus-mCherry-GFP-LC3B and JC1 assay kit was obtained from Beyotime (China). riboFECT CP Transfection Kit (China) and jetPRIME Transfection kit (France) were purchased from Ribo and Polyplus, respectively. Primary antibodies: TFRC, SLC11A2, IREB1, IREB2, NCOA4, Ferritin, 4-HNE, MDA, LC3B, TOMM20, MTCO1, p-Tau, β-Amyloid (Abcam, USA); SLC40A1 (Novus, USA); cGAS, p-STING, STING, p-TBK1, TBK1, p-NF-κB, NF-κB, p-IRF3, IRF3, P21, TNF-α, IL-6, IFN-β, p-H2AX (CST, USA); 8-OHdG, Rhodopsin, Arrestin, dsDNA (Santa, USA); SDHB, NDUFB8, ATP5A1, UQCRC2, β-actin (Proteintech, China). Information of reagents and antibodies is provided in Supplementary Tables 1 and 2.

### Cell culture

The human retinal epithelial cells (ARPE-19, Zhong Qiao Xin Zhou Biotechnology, Shanghai, China) was cultured in DMEM/F12 medium contain 10% FBS (Gibco, USA), 100 U/mL penicillin and 100 μg/mL streptomycin at 37 °C, 5% CO_2_. ARPE-19 cells were treated with TBH (100 μM) for 48 h to induce cell senescence and FAC (200 μM) for 48 h to mimic intracellular accumulation of iron. The cells were pretreated with different small molecule inhibitors, including DFO (20 μM), Mito-T (10 μM), 3-MA (5 mM), CQ (5 μM), Baf-A1(100 nM) and H-151 (10 μM) for further explore the underlying mechanisms.

### Animals

Male C57BL/6J mice (C57, 8 weeks, 22–25 g, Changzhou Cavans Experimental Animal Co., Ltd, China) were used. Animals were housed in 12 h light/dark cycle with food and water ad libitum. All animal experiments were performed according to the Guide for the Care and Use of Laboratory Animals of the National Institute of Health and approved by the Animal Care and Use Committee of Nanjing Medical University (2022-44).

The mice were randomly divided into D-gal treated group for aging model and FAC treated group for iron overload retinal function model. The D-gal-treated group was further divided into three subgroups (*n* = 15): control group, D-gal-induced group, and D-gal + DFO-treated group. The FAC-treated group was divided into three subgroups (*n* = 15): control group, FAC-induced group and FAC + DFO-treated group (D-gal, 200 mg/kg, once per day, hypodermic injection; FAC, 40 mg/kg, every 3 days, intraperitoneal injection; DFO, 100 μM, 1 μL/per eye, every 4 weeks, intravitreal injection). All mice were sacrificed after 16 weeks, and samples of eyeballs were taken for further experiments.

### Intravitreal injection

Inhalational anesthesia (3.5% isoflurane) was performed on mice before intravitreal injection (IVI). Mice were placed under the operating microscope using a 10 µl Hamilton syringe (Hamilton, 7803–05) to perform IVI. The tip of the syringe was inserted at a 45° angle into the eye and passed through the sclera to the vitreous body without damaging the lens or retina [63]. DFO (100 μM, 1 μL/per eye) was injected into the vitreous cavity every 4 weeks for 16 weeks. After IVI, ofloxacin ointment was applied to the eyeball for protection.

### Retinal imaging

The optical coherence tomography (OCT) system (Micron IV; Phoenix Research Labs, Pleasanton, CA, USA) was used to take live-fundus photographs of mice.

### Transient knockdown and overexpression

ARPE-19 cells were plated into six-well plates to transfection. ARPE-19 transient knockdown was performed by using TFRC or NCOA4 siRNA (riboFECT CP Transfection Kit (RiboBio, China)) at 80% confluence. Briefly, 120 μL Opti-MEM medium containing 12 μL riboFECT CP, 100 nM TFRC/NCOA4 siRNA or 100 nM NC was premixed in a 6-well plate. After 48 h, the transfection efficiency was assessed by Western blot.

ARPE-19 transient overexpression of Ferritin using pcDNA3.1/FTH1 (RiboBio, China). Firstly, 2 ug FTH1 cDNA was added to 200 uL jetPRIME® buffer, then 4 uL jetPRIME® was added, vortexed and mixed well, and then incubated for 10 min at room temperature. Finally, the transfection complex was added dropwise to the cells. After 48 h, the overexpression protein levels were detected by Western blot. Detailed information is provided in Supplementary Table 3.

### Senescence-associated-β-galactosidase (SA-β-gal) assay

ARPE-19 cells and the frozen sections of mice were stained with the SA-β-Ga Staining Kit (C0602, Beyotime). Briefly, cultured cells and sections were fixed for 15 min and then incubated with SA-β-gal staining reagent at 37 °C overnight. The images of stained-positive cells were counted under light microscopy (200×, Olympus, Japan). The percentage of senescent cells was determined by the ratio of blue (SA-β-Gal-positive) cells to total cells obtained from 5 random view fields of each sample.

### Measurement of Fe^2+^

To detect intracellular and mitochondrial Fe^2+^ levels, FerroOrange (F374, Dojindo) and Mito-FerroGreen (M489, Dojindo) were used. Briefly, ARPE-19 cells were washed with PBS and stained with 1 μM FerroOrange or 5 μM Mito-FerroGreen for 30 min at 37 °C. Images were acquired using Laser Scanning Confocal Microscopy (40×, Leica, Germany). The fluorescence intensity was measured by ImageJ. Lillie’s Ferrous Iron stain (G3320, Solarbio) was conducted to evaluate retinal Fe^2+^ levels in paraffin-embedded tissue (4 μm-thick). Images were acquired under a light microscope (200×, Olympus, Japan).

### Detection of lipid peroxidation and ROS

To detect lipid peroxidation levels, cells were stained with C11-BODIPY (581/591) probes (10 μM, D3681, Thermo Fisher). After incubation for 30 min at 37 °C, ARPE19 cells were washed with PBS, observed under Laser scanning Confocal Microscopy (40×, Leica, Germany), and quantified using ImageJ software. Lipid peroxidation was examined by fluorescence change from red to green (r-Bodipy/o-Bodipy). Measurement of mitochondrial superoxide in ARPE19 cells was conducted using MitoSOX Red (5 μM, M36008, Thermo Fisher). Mito-Tracker Red (200 nM, C1035, Beyotime) and Mito-Tracker Green (200 nM, C1048, Beyotime) were used to co-stain the mitochondria. Images were obtained under Laser scanning Confocal Microscopy (40×, Leica, Germany).

### Detection of autophagic flux

mCherry-GFP-LC3B is a fluorescent protein for observing the level of autophagic flux. AdPlus-mCherry-GFP-LC3B (C3012, Beyotime) was used to assess the autophagic flux in ARPE19. The red fluorescence of the cells in the images can be used to indicate the localization of the fusion protein in the lysosome or autolysosome, indicating the activation of autophagic flux [64]. ARPE-19 were seeded in 24 well-plates and infected with adenovirus for 24 h, followed by image collection by Laser scanning Confocal Microscopy (40×, Leica, Germany).

### Detection of mitochondrial membrane potential

Mitochondrial membrane potential was measured using a JC-1 assay kit (C2006, Beyotime). The ARPE19 cells were stained with JC-1 dye for 20 min at 37 °C; after washing with dilution buffer three times, the cells were imaged under Laser scanning Confocal Microscopy (40×, Leica, Germany). The mitochondrial membrane potential was calculated by the ratio of red to green fluorescence (aggregates/monomers).

### Hematoxylin and eosin (H&E) staining

For the HE staining, Paraffin sections of retinal (4 μm) were dehydrated using graded ethanol (50, 75, 80, 90, 95, and 100%), and then stained with hematoxylin and eosin kit (AR1180, BOSTER). The images were captured using a light microscopy (200×, Olympus, Japan).

### Immunohistochemistry (IHC) and immunofluorescence (IF)

IHC staining was carried out with Paraffin slides (4 μm). The Slides were dewaxed in xylene and hydrated in ethanol, and treated in 3% H_2_O_2_ for 30 min, followed by microwave treatment in 10 mM citrate buffer for 15 min and blocked with 5% BSA for 1 h, then incubated with: 8-OhdG (1:50), MDA (1:200), 4-HNE (1:200), Aβ (1:400), p-tau (1:500), overnight at 4 °C. The next day, Slides were incubated with biotinylated goat anti-mouse/rabbit IgG for 45 min followed by incubation with DAB for 2–3 min. Counterstaining was performed with Mayer’s hematoxylin. Images were acquired using a light microscopy (200×, Olympus, Japan).

For IF staining, Cells or frozen sections were fixed with Fixative solution for 15 min, permeabilized with 0.1% Triton-100 for 30 min and blocked with 5% BSA for 1 h. The samples were incubated with p-H2AX (1:400), 8-OhdG (1:50), MDA (1:200), 4-HNE (1:200), TOMM20 (1:250), dsDNA (1:100), Rhod (1:100), Arrestin (1:100) overnight at 4 °C and then incubated with Alexa Fluor 594-conjugated secondary antibody (1: 400, A11012, Thermo Fisher, Waltham, MA, USA) for 2 h. After staining with DAPI (10 min) the samples were observed using Laser scanning Confocal Microscopy (40×, Leica, Germany).

### Western blotting

The ARPE-19 and tissues were lysed at 4 °C with RIPA buffer (Beyotime, P0013B) containing protease, phosphatase inhibitors and PMSF (1 mM, Beyotime, ST506). Protein concentration was determined using the BCA Protein Assay Kit (Beyotime, P0012S). Samples were transferred to PVDF membranes after 10% SDS/PAGE electrophoresis. Blocked in 5% skim milk for 2 h and incubated with primary antibodies: TFRC (1:1000), SLC11A2 (1:1000), IREB1 (1:1000), IREB2 (1:1000), NCOA4 (1:1000), FTH1(1:200), LC3B (1:2000), MTCO1 (1:1000), SLC40A1 (1:1000), cGAS (1:1000), p-STING (1:1000), STING (1:1000), p-TBK1 (1:1000), TBK1 (1:1000), p-NF-κB (1:1000), NF-κB (1:1000), p-IRF3 (1:1000), IRF3 (1:1000), P21 (1:1000), TNF-α (1:1000), IL-6 (1:1000), IFN-β (1:1000), p-H2AX (1:1000), SDHB (1:5000), NDUFB8 (1:5000), ATP5A1 (1:2000), UQCRC2 (1:2000). Protein bands were measured using an enhanced chemiluminescence kit (34580, Thermo Fisher) after combining with horseradish peroxidase (HRP)-linked antibodies for 2 h.

### Statistical analysis

All experiments were conducted at least three times. ImageJ software (NIH) was used for quantitative data analysis. Statistical analysis was performed with GraphPad Prism (GraphPad Software version 8.0, San Diego, CA) using the Student’s two-tailed *t* test or one-way ANOVA with Tukey’s honestly significant difference test. Data were expressed as mean ± SD. *p* value < 0.05 was considered statistically different.

### Supplementary information

Supplementary Material

Full and uncropped western

## Data Availability

For all data requests, please contact the corresponding author.
