# Supplementary material for: Iron derived from NCOA4-mediated ferritinophagy causes cellular senescence via the cGAS-STING pathway
Source: Cell Death Discov. 2023 Nov 18;9:419. doi: 10.1038/s41420-023-01712-7 (PMC10657394; doi:10.1038/s41420-023-01712-7)

**SUPPLEMENTARY MATERIAL**

**Table 1. Main materials and reagents**

| **Reagents** | **Source** | **Identifier** |
| --- | --- | --- |
| Tert-butyl hydroperoxide | Sigma | 458139 |
| Ferric ammonium citrate | Selleck | E0375 |
| D-galactose | Aladdin | G100369 |
| Deferoxamine mesylate | MCE | HY-B0988 |
| Mito-TEMPO | MCE | HY-112879 |
| 3-Methyladenine | MCE | HY-19312 |
| Chloroquine | MCE | HY-17589A |
| Bafilomycin A1 | MCE | HY-100558 |
| H-151 | MCE | HY-112693 |
| FerroOrange | Dojindo | F374 |
| Mito-FerroGreen | Dojindo | M489 |
| MitoPeDPP | Dojindo | M466 |
| C11-BODIPY (581/591) probes | Thermo Fisher | D3861 |
| MitoSOX™ Red | Thermo Fisher | M36008 |
| Lillie’s Ferrous Iron stain | Solarbio | G3320 |
| AdPlus-mCherry-GFP-LC3B | Beyotime | C3012 |
| SA-β-Gal Staining Kit | Beyotime | C0602 |
| Mito-Tracker Green | Beyotime | C1048 |
| Mito-Tracker Red | Beyotime | C1035 |
| JC1 assay kit | Beyotime | C2006 |
| Penicillin-Streptomycin solution | Beyotime | C0222 |
| RIPA lysis buffer | Beyotime | P0013B |
| HE staining kit | BOSTER | AR1180 |
| IHC staining kit | BOSTER | SV0004 |
| DAB | BOSTER | AR1027 |
| [Fetal Bovine Serum](https://www.thermofisher.com/cn/zh/home/life-science/cell-culture/mammalian-cell-culture/fbs.html?SID=fr-fbs-main) | Gibco | 10099-141 |
| Trypsin-EDTA (0.25%) | Gibco | 25200072 |
| DMEM/F-12 | Gibco | 11330057 |
| riboFECT CP Transfection Kit | Ribo | C10511-1 |
| jetPRIME Transfection kit | Polyplus | PT-114-15 |
| Goat Anti-Mouse IgG(H+L) | Jackson | 115-035-003 |
| Goat Anti-Rabbit IgG (H+L) | Jackson | 111-035-003 |
| Goat anti-Mouse IgG (H+L), Alexa Fluor 555 | Thermo Fisher | A-21422 |
| Goat anti-Rabbit IgG (H+L), Alexa Fluor 555 | Thermo Fisher | A-21428 |
| Goat anti-Mouse IgG (H+L), Alexa Fluor 488 | Thermo Fisher | A-11001 |
| DAPI | Thermo Fisher | R37606 |
| ECL kit | Thermo Fisher | 34580 |

**Table 2. Primary antibodies**

| **Primary antibodies** | **Source** | **Identifier** |
| --- | --- | --- |
| Rabbit monoclonal anti-TFRC | Abcam | ab214039 |
| Mouse monoclonal anti-SLC11A2 | Abcam | ab55735 |
| Rabbit monoclonal anti-IREB1 | Abcam | ab183721 |
| Rabbit monoclonal anti-IREB2 | Abcam | ab181153 |
| Rabbit polyclonal anti-NCOA4 | Abcam | ab86707 |
| Rabbit monoclonal anti-Ferritin | Abcam | ab75973 |
| Rabbit polyclonal anti-4-HNE | Abcam | ab46545 |
| Rabbit polyclonal anti-MDA | Abcam | ab27642 |
| Rabbit monoclonal anti-LC3B | Abcam | ab192890 |
| Rabbit monoclonal anti-TOMM20 | Abcam | ab186735 |
| Rabbit monoclonal anti-MTCO1 | Abcam | ab203912 |
| Rabbit monoclonal anti-p-Tau | Abcam | ab109390 |
| Rabbit monoclonal anti-β-Amyloid | Abcam | ab201060 |
| Rabbit polyclonal anti-SLC40A1 | Novus | NBP1-21502 |
| Rabbit monoclonal anti-cGAS | CST | #15102 |
| Rabbit monoclonal anti-p-STING | CST | #50907 |
| Rabbit monoclonal anti-STING | CST | #13647 |
| Rabbit monoclonal anti-p-TBK1 | CST | #5483 |
| Rabbit monoclonal anti-TBK1 | CST | #3504 |
| Rabbit monoclonal anti-p-NF-κB | CST | #3033 |
| Rabbit monoclonal anti-NF-κB | CST | #8242 |
| Rabbit monoclonal anti-p-IRF3 | CST | #29047 |
| Rabbit monoclonal anti-IRF3 | CST | #11904 |
| Rabbit monoclonal anti-p21 | CST | #2947 |
| Rabbit monoclonal anti-TNF-α | CST | #6945 |
| Rabbit monoclonal anti-IL-6 | CST | #12153 |
| Rabbit monoclonal anti-IFN-β | CST | #73671 |
| Rabbit monoclonal anti-p-H2AX | CST | #9718 |
| Mouse monoclonal anti-8-OHdG | Santa | sc-393871 |
| Mouse monoclonal anti-Rhodopsin | Santa | sc-57432 |
| Mouse monoclonal anti-Arrestin | Santa | sc-271159 |
| Mouse monoclonal anti-dsDNA | Santa | sc-58749 |
| Rabbit polyclonal anti-SDHB | Proteintech | 10620-1-AP |
| Rabbit polyclonal anti-NDUFB8 | Proteintech | 14794-1-AP |
| Rabbit polyclonal anti-ATP5A1 | Proteintech | 14676-1-AP |
| Rabbit polyclonal anti-UQCRC2 | Proteintech | 14742-1-AP |
| Mouse monoclonal anti-β-actin | Proteintech | 66009-1-Ig |

**Table 3. The sequences of the siRNA**

| **Product number** | **Product name** | **Sequence (5’–3’)** |
| --- | --- | --- |
| siG000008031A | si-NCOA4-101 | GGCTCATGCTAGTTCAGCA |
| siG000008031B | si-NCOA4-102 | GCTCATGCTAGTTCAGCAA |
| siG000008031C | si-NCOA4-103 | GAAGTGGTTATATCGAACT |
| stB0002649A | si-TFRC-001 | GTAGGATGGTAACCTCAGA |
| stB0002649B | si-TFRC-002 | GCACAGCTCTCCTATTGAA |
| stB0002649C | si-TFRC-003 | GGAGACTTCTTCCGTGCTA |
| siB161011044323 | NC-siRNA | GGCTCTAGAAAAGCCTATGC |

**Table 4. Basic information of the corresponding GEO data set**

| **Group** | **Accession** | **Tissue** | **Gender** | **Age** |
| --- | --- | --- | --- | --- |
| Young | GSM1401927 | Whole blood | Females | 20 |
| Young | GSM1401936 | Whole blood | Females | 21 |
| Young | GSM1402039 | Whole blood | Females | 19 |
| Young | GSM1402073 | Whole blood | Females | 20 |
| Young | GSM1402084 | Whole blood | Females | 25 |
| Young | GSM1401959 | Whole blood | Males | 24 |
| Young | GSM1401980 | Whole blood | Males | 18 |
| Young | GSM1402009 | Whole blood | Males | 21 |
| Young | GSM1402021 | Whole blood | Males | 21 |
| Young | GSM1402152 | Whole blood | Males | 25 |
| Old | GSM1401921 | Whole blood | Females | 59 |
| Old | GSM1401973 | Whole blood | Females | 55 |
| Old | GSM1401995 | Whole blood | Females | 61 |
| Old | GSM1401996 | Whole blood | Females | 59 |
| Old | GSM1402140 | Whole blood | Females | 62 |
| Old | GSM1402011 | Whole blood | Males | 58 |
| Old | GSM1402031 | Whole blood | Males | 63 |
| Old | GSM1402059 | Whole blood | Males | 57 |
| Old | GSM1402087 | Whole blood | Males | 65 |
| Old | GSM1402133 | Whole blood | Males | 56 |

**FIGURE LEGENDS**

**Fig. S1 The Boxplot depicting the read counts normalization and the distributions of age.** **A** Rows represent samples and columns represent the gene expression values in the samples. **B** The age distribution of the samples.

**Fig. S2 Accumulation of Aβ and p-tau in D-gal-induced mice retinal and brain tissue. A, B** Immunohistochemistry of Aβ and p-tau in mice retinal and brain tissue. Scale bars, 100 μm.

**Fig. S3 ARPE-19 cells were transduced with TFRC and NCOA4 siRNA. A** ARPE-19 cells were transduced with TFRC and NCOA4 siRNA for 48 hrs, and the transfection efficiency was assessed by Western blot.

**Fig. S4 Detection of lipid peroxidation levels. A, B** lipid peroxidation assessment in cell was detected by C11-BODIPY probes (10 μM, 30 min), reduced (red) and oxidized (green) Bodipy (n = 3). Scale bars, 25 μm. Mean ± SD. *P < 0.05 vs. control group. #P < 0.05 vs. TBH or FAC group.

**Fig. S5 Five mitochondrial functions-associated genes were negatively correlated with NCOA4. A** COX4I1, PHB, SDHA, SOD1 and TIMM44 was negatively correlated with NCOA4, respectively, in the collected 20 human blood samples.

**Fig. S6 Detection of ferritinophagy levels.** A The protein content of NCOA4 and Ferritin was assessed by Western blot (n=3). The data were expressed as mean ± SD. *P < 0.05 vs. control group. #p<0.05 vs. TBH group.

**Fig. S7 Accumulation of Aβ and p-tau in FAC-induced mice retinal and brain tissue. A, B** Immunohistochemistry of Aβ and p-tau in mice retinal and brain tissue. Scale bars, 100 μm.

**Fig. S8 Detection of lipid peroxidation metabolites.** ARPE-19 cells were overexpressed ferritin by FTH1 cDNA transfection. ARPE-19 cells were transduced with NCOA4 siRNA for 48 hrs. **A, B** Immunofluorescence staining of 4-HNE and MDA. Scale bars, 25 μm.

**Fig. S9 Detection of mitochondrial iron. A** Mito-FerroGreen (5 μM, 30 min) and MitoTracker-Red (200 nM, 30 min) was used to co-stained mitochondria iron and mitochondria. Scale bars, 25 μm.

**Supplementary Figure. S1**


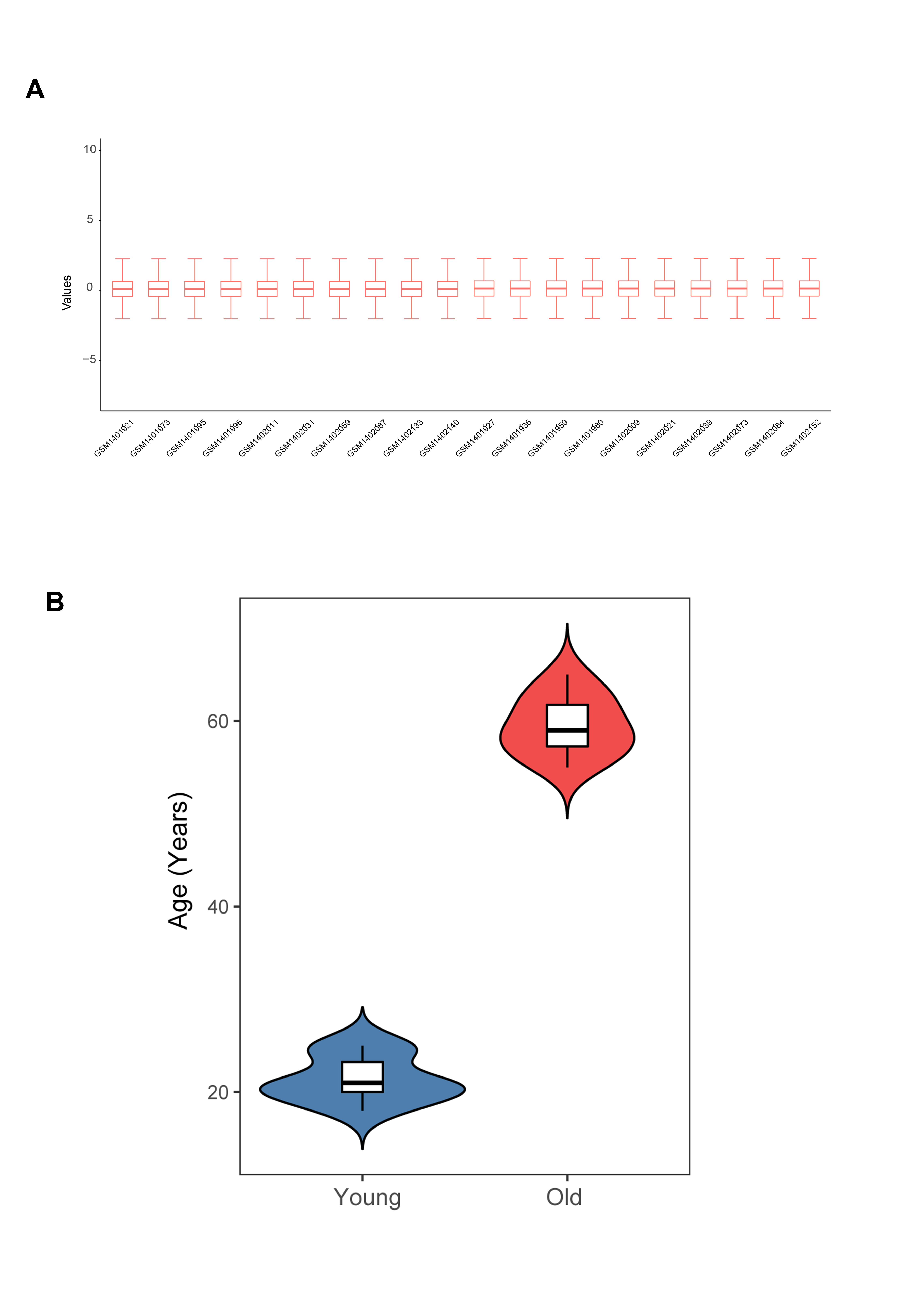


**Supplementary Figure. S2**


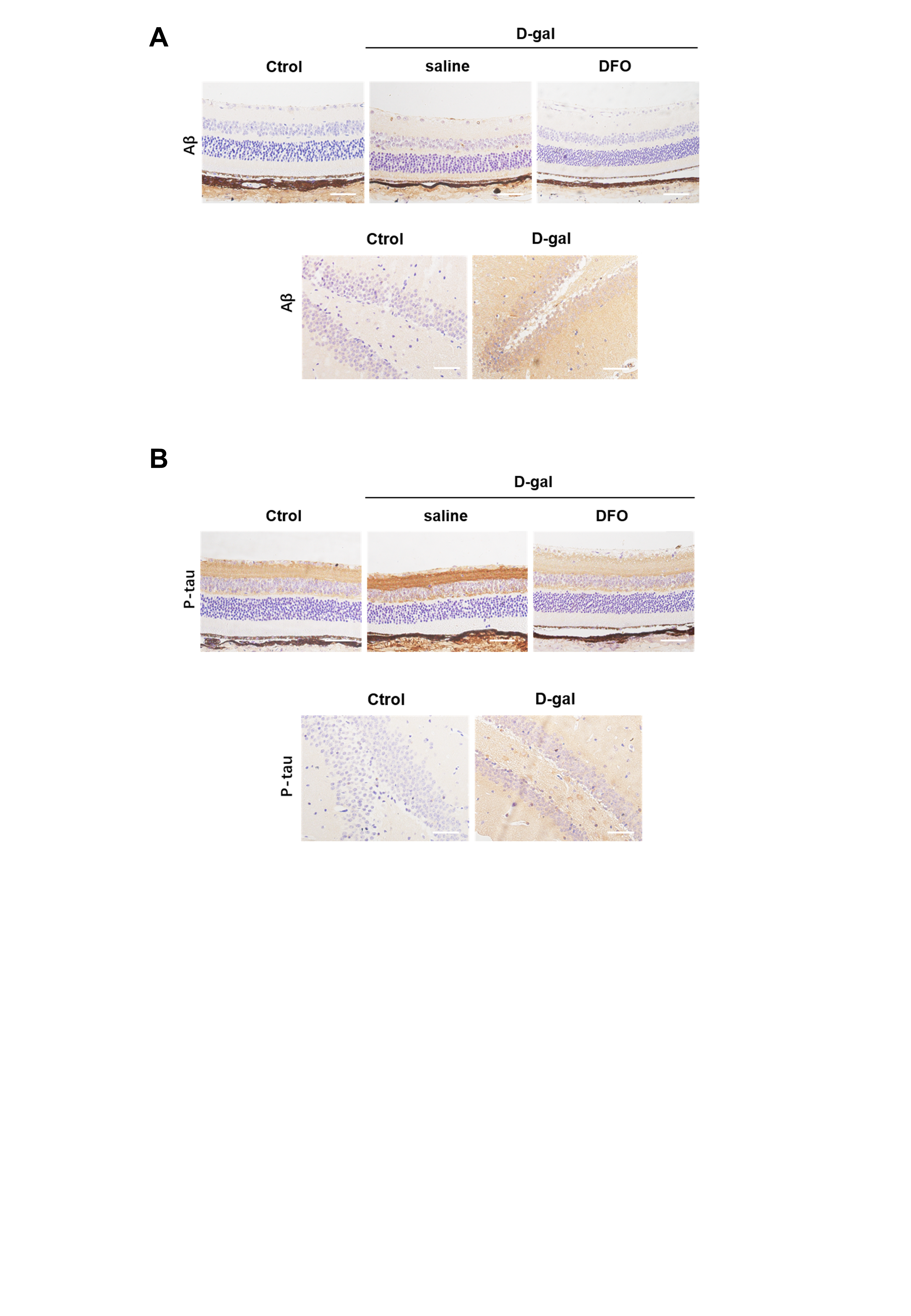


**Supplementary Figure. S3**


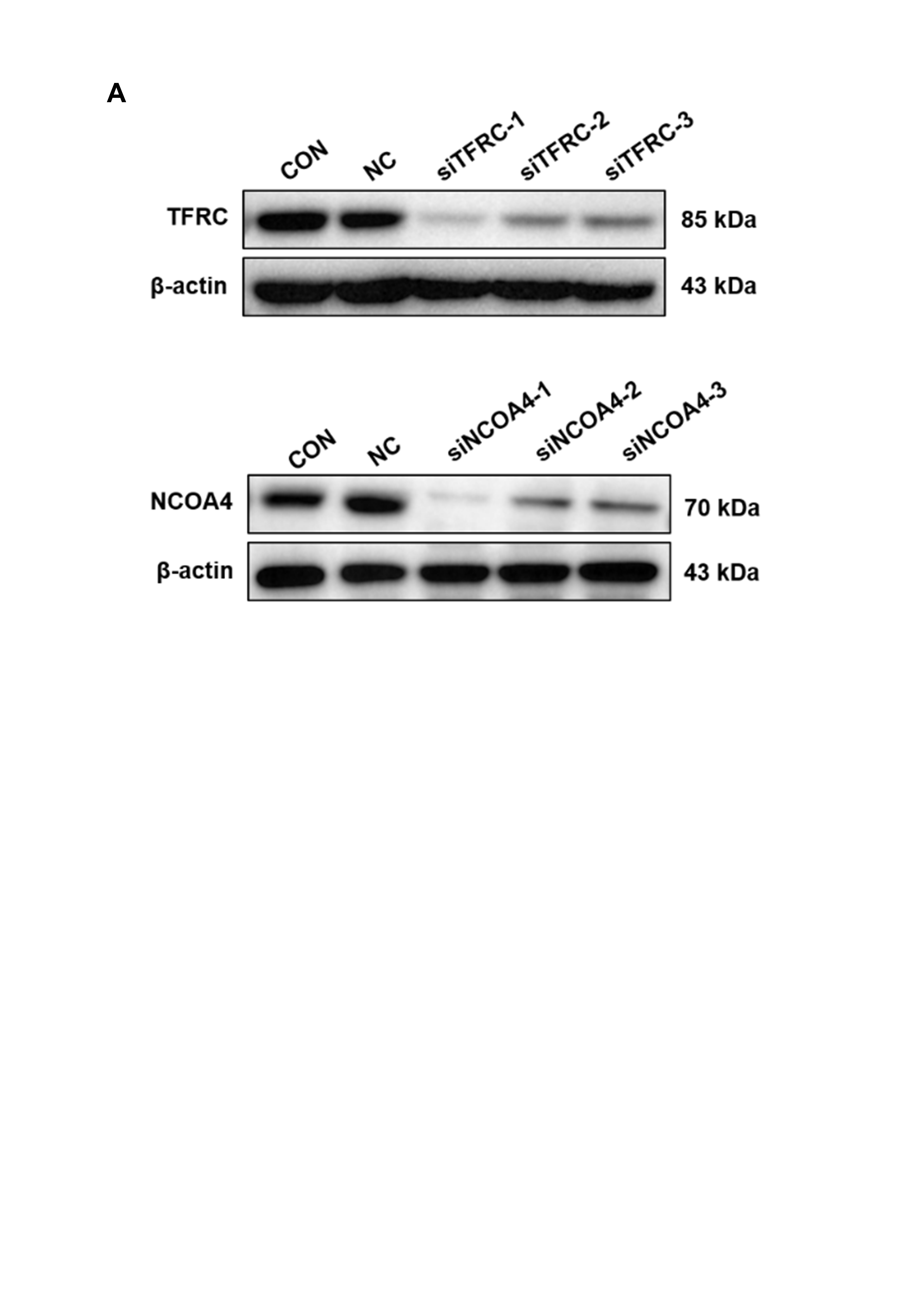


**Supplementary Figure. S4**


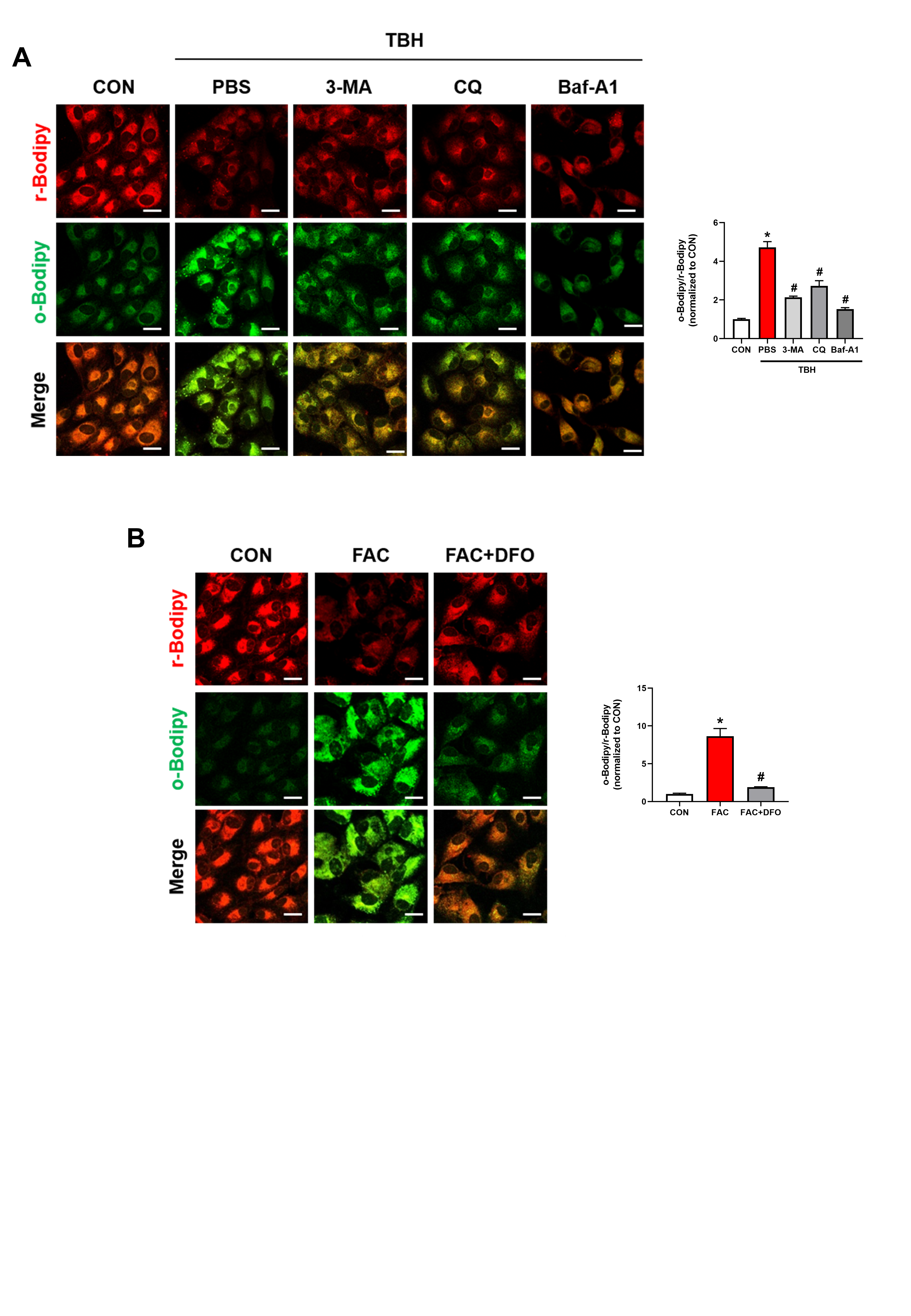


**Supplementary Figure. S5**


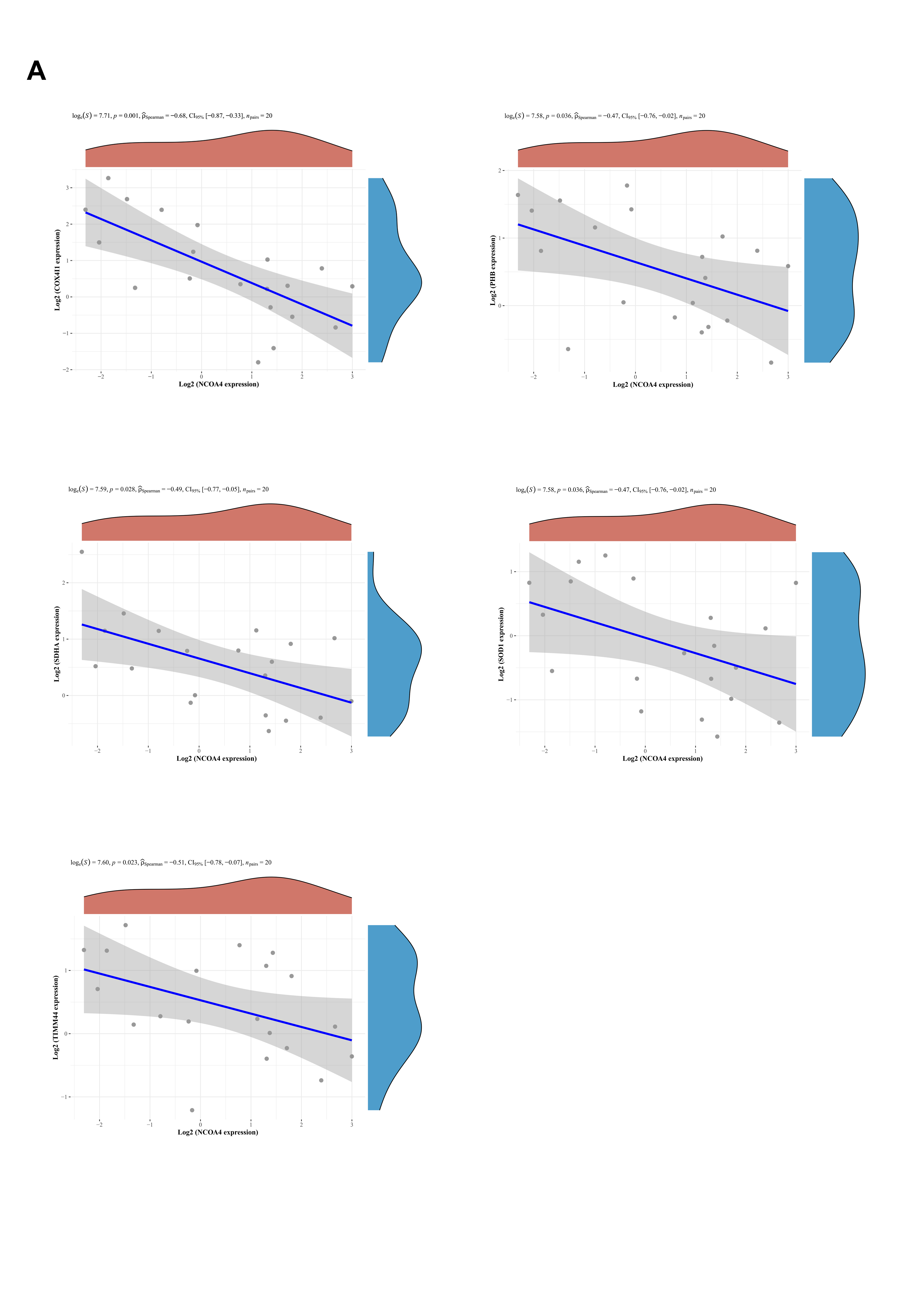


**Supplementary Figure. S6**

**
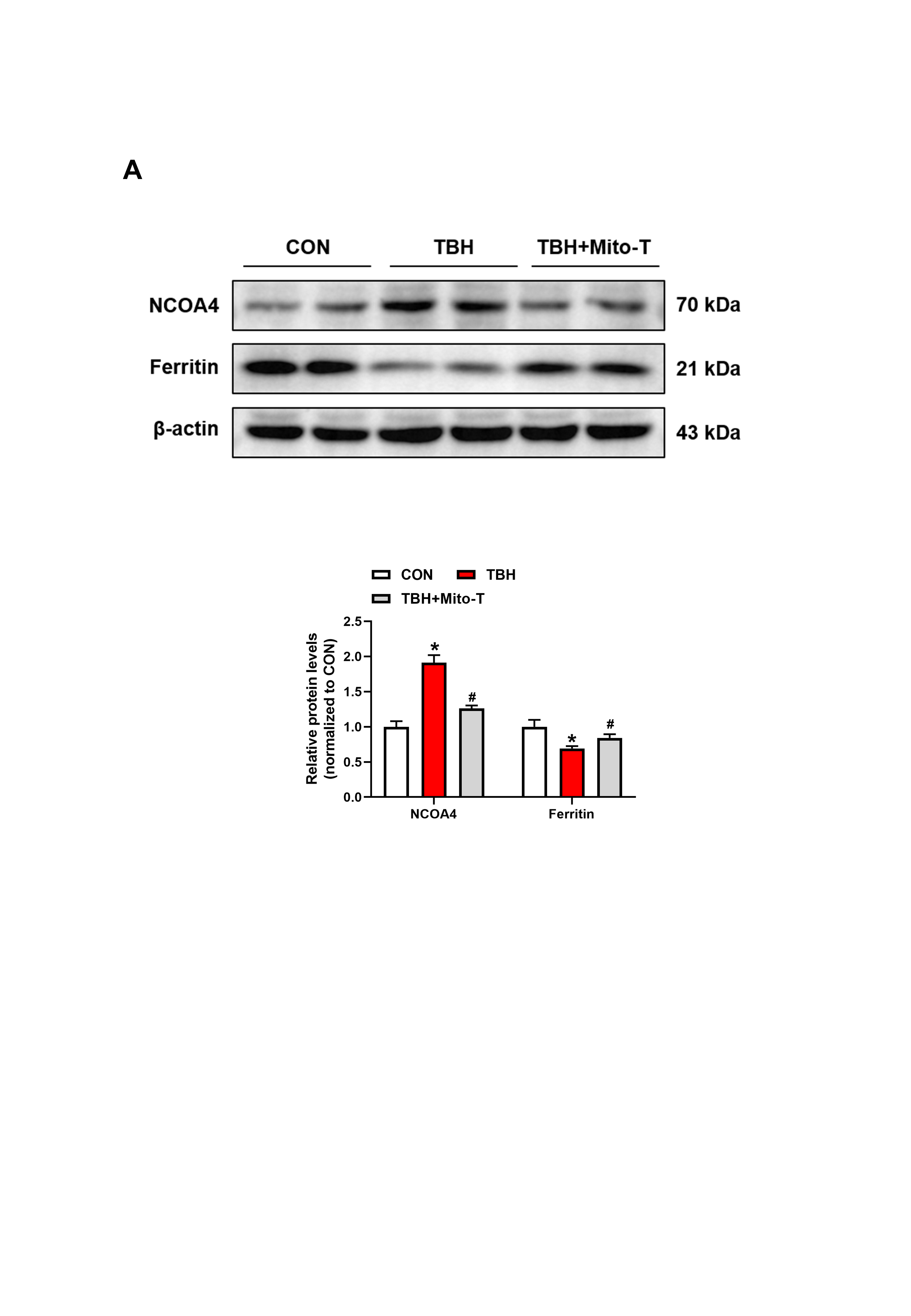
**

**Supplementary Figure. S7**


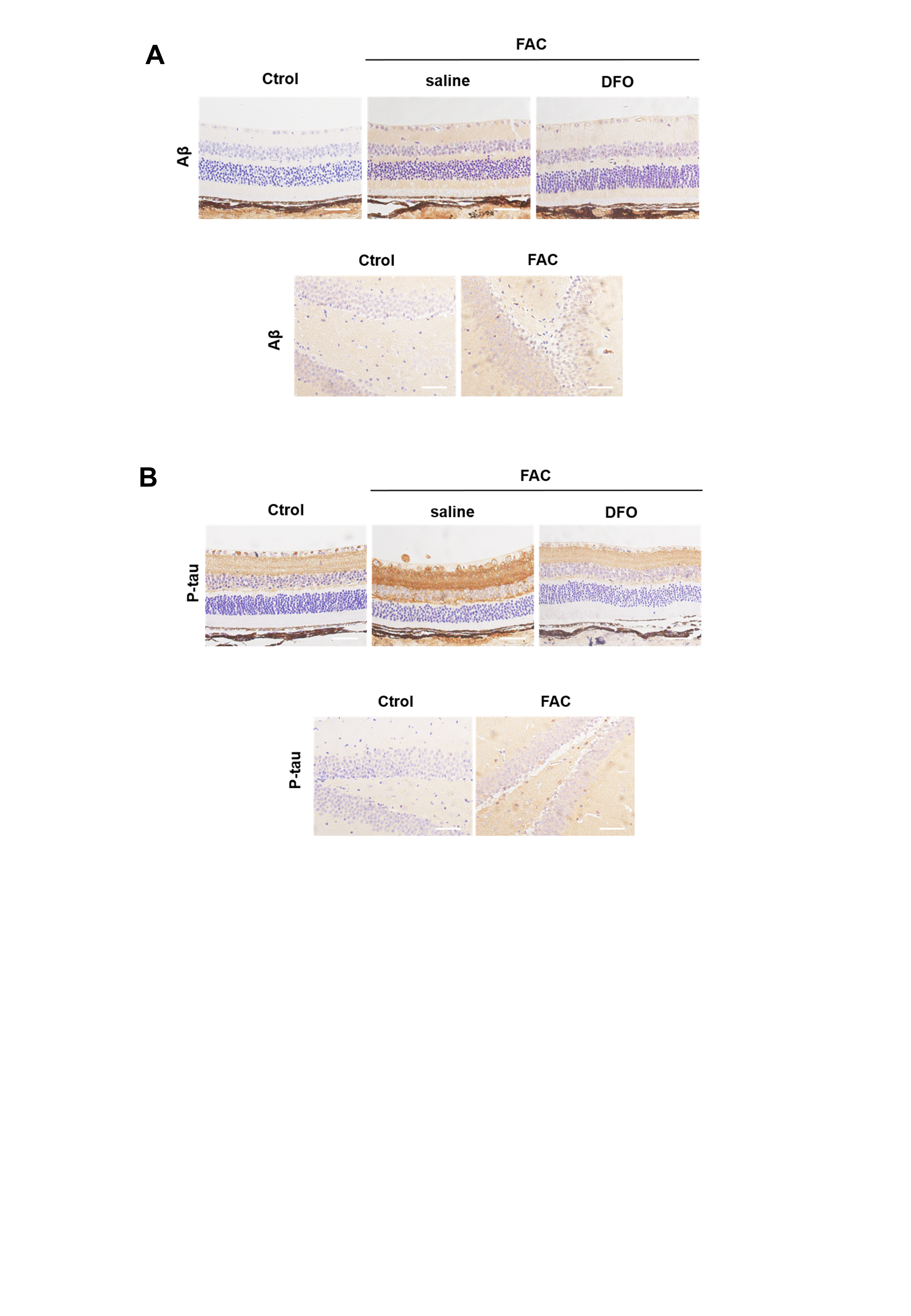


**Supplementary Figure. S8**


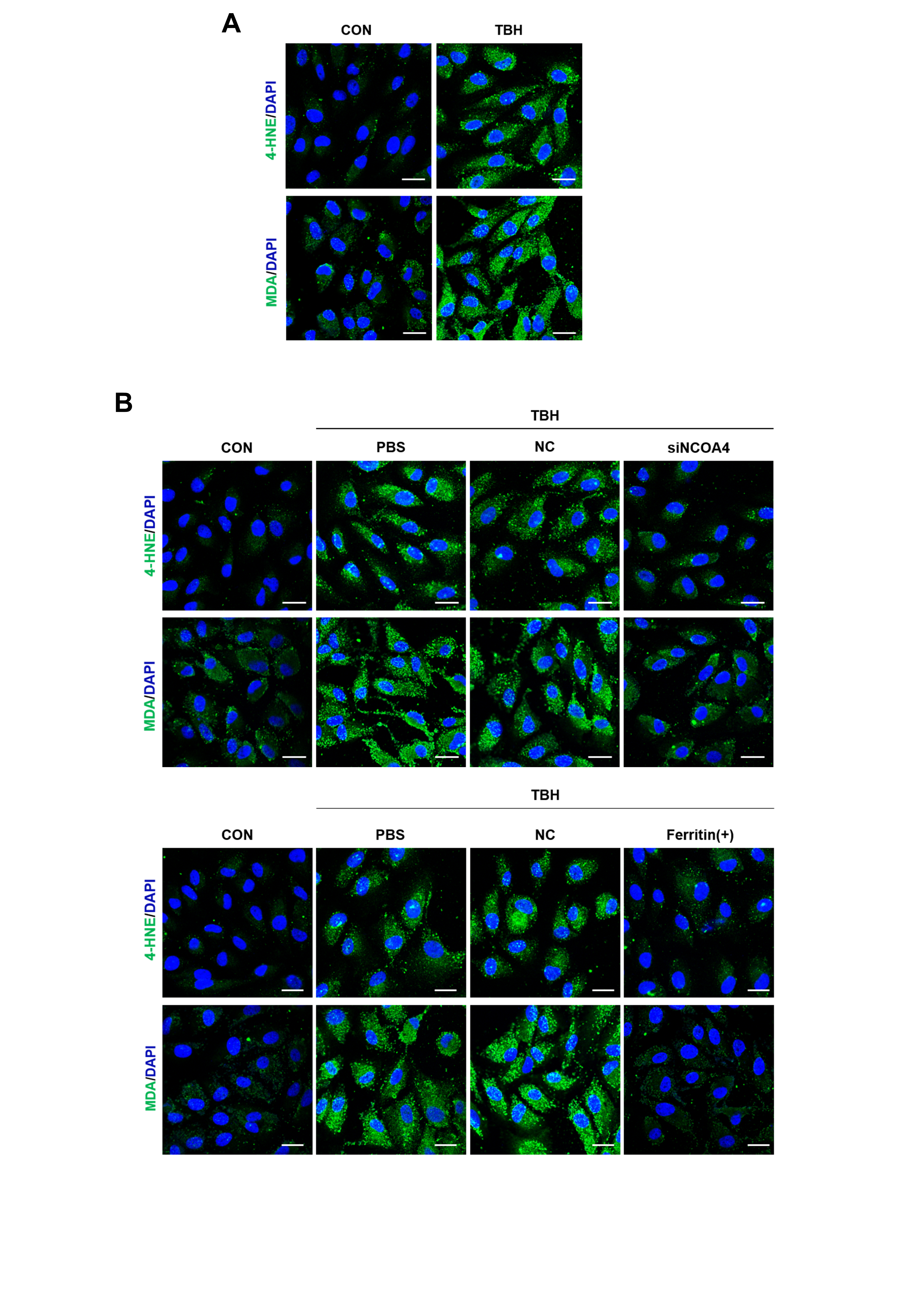


**Supplementary Figure. S9**


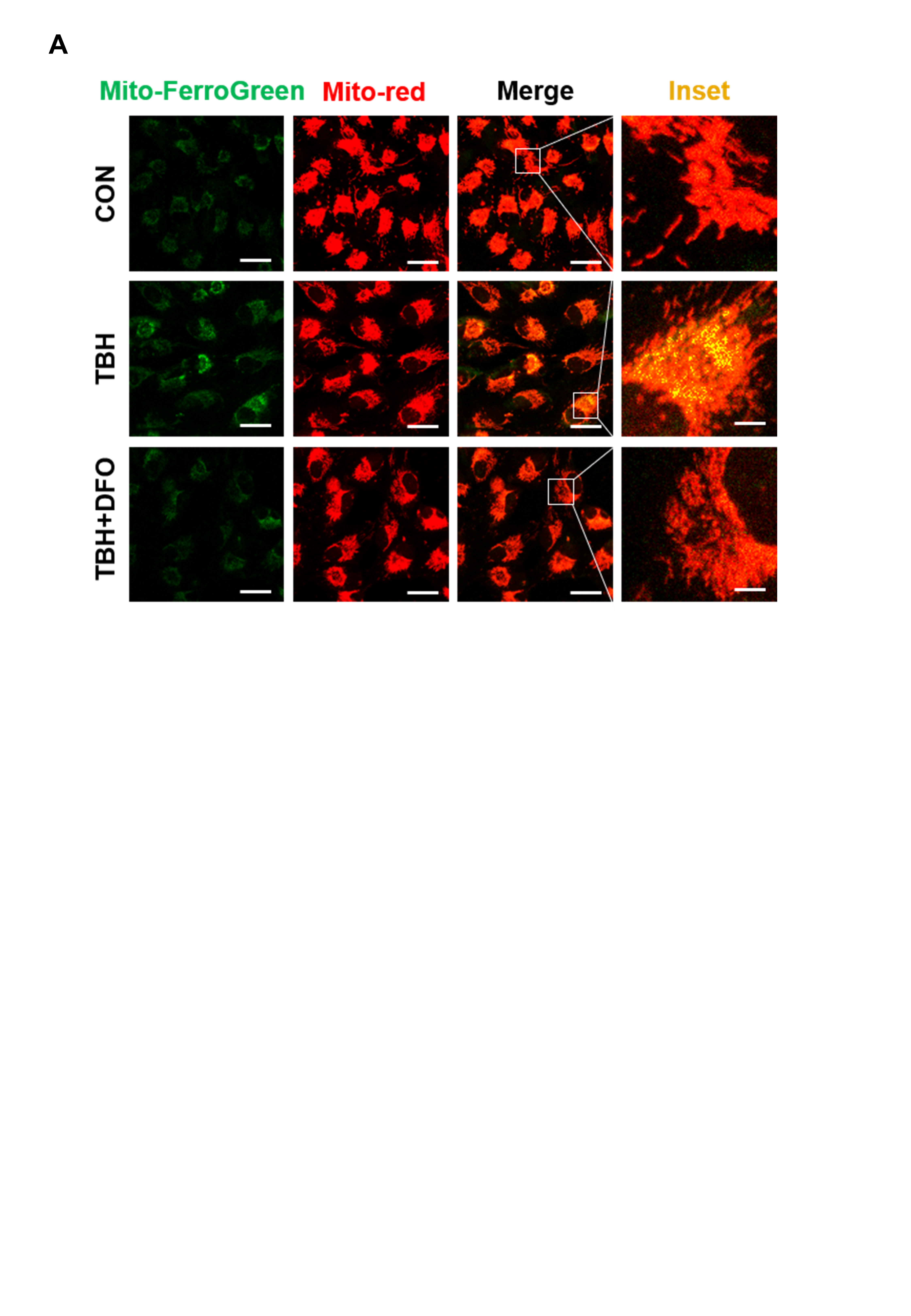

Supplement: Supplementary file 1 — Supplementary Material [file 41420_2023_1712_MOESM1_ESM.docx]
