# Supplementary material for: Iron derived from NCOA4-mediated ferritinophagy causes cellular senescence via the cGAS-STING pathway
Source: Cell Death Discov. 2023 Nov 18;9:419. doi: 10.1038/s41420-023-01712-7 (PMC10657394; doi:10.1038/s41420-023-01712-7)
Supplement: Supplementary file 2 — Full and uncropped western [file 41420_2023_1712_MOESM2_ESM.pdf]

Figure 1H

Ctrol D-gal

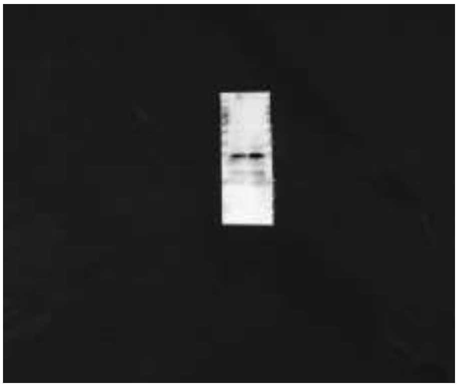

IFN-β

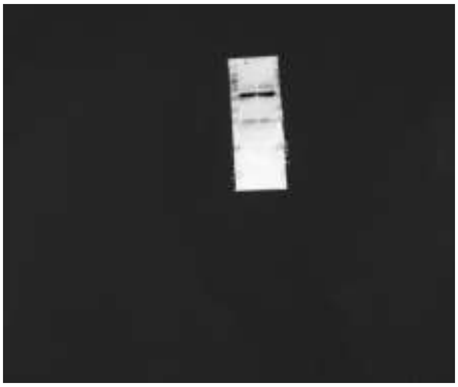

β-actin

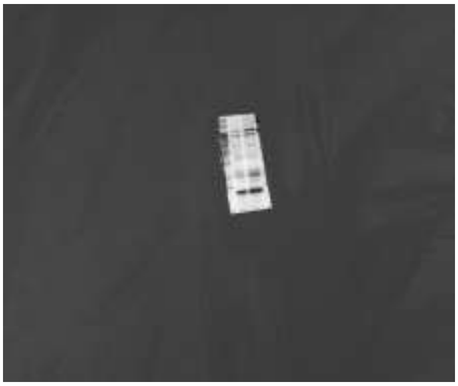

TNF-α

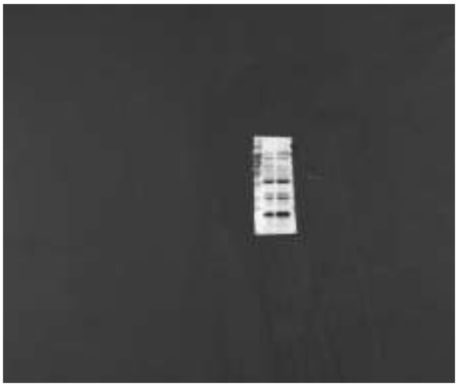

β-actin

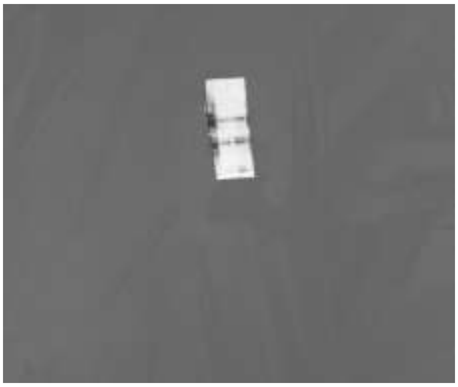

IL-6

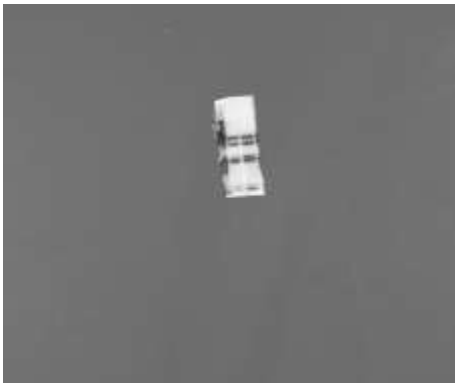

β-actin

Figure 2B

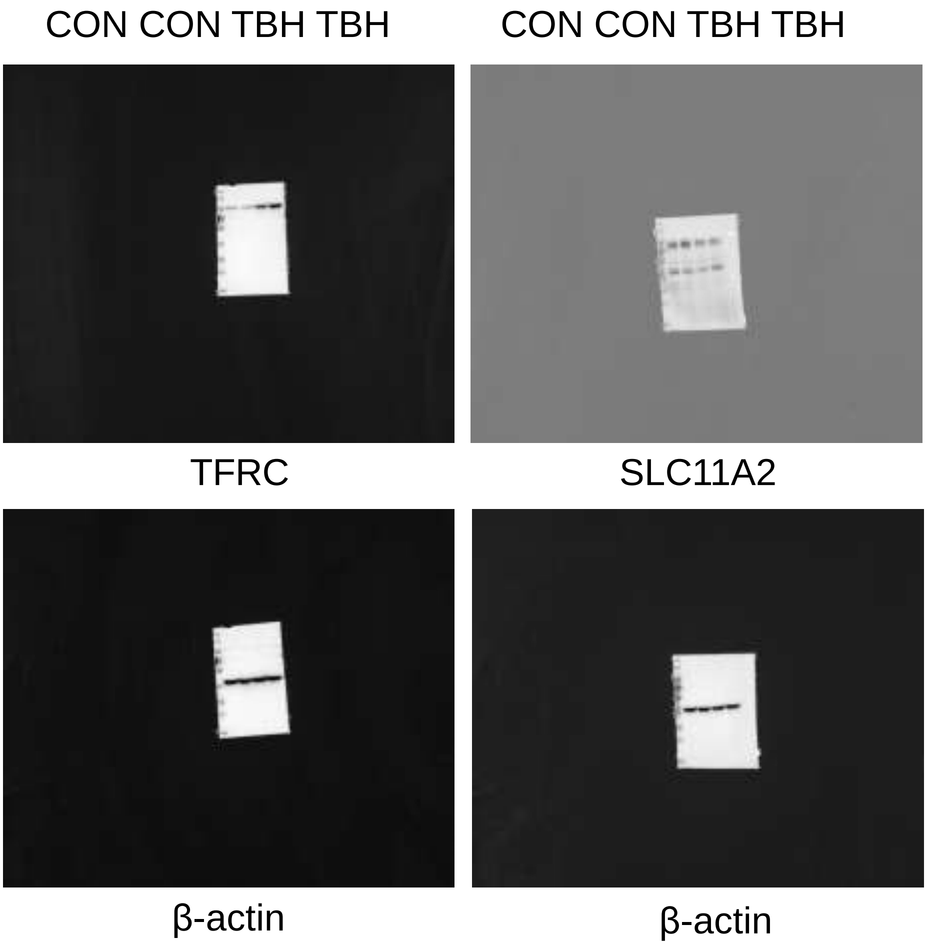

Figure 2B

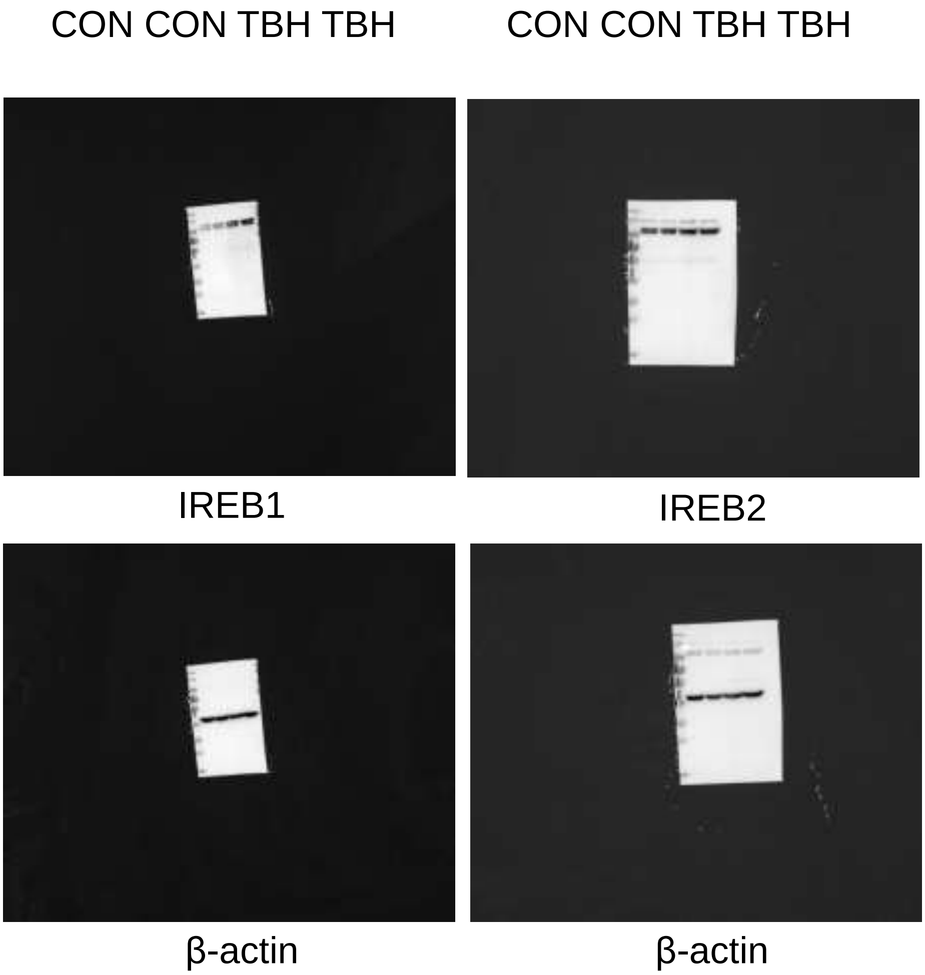

Figure 2B

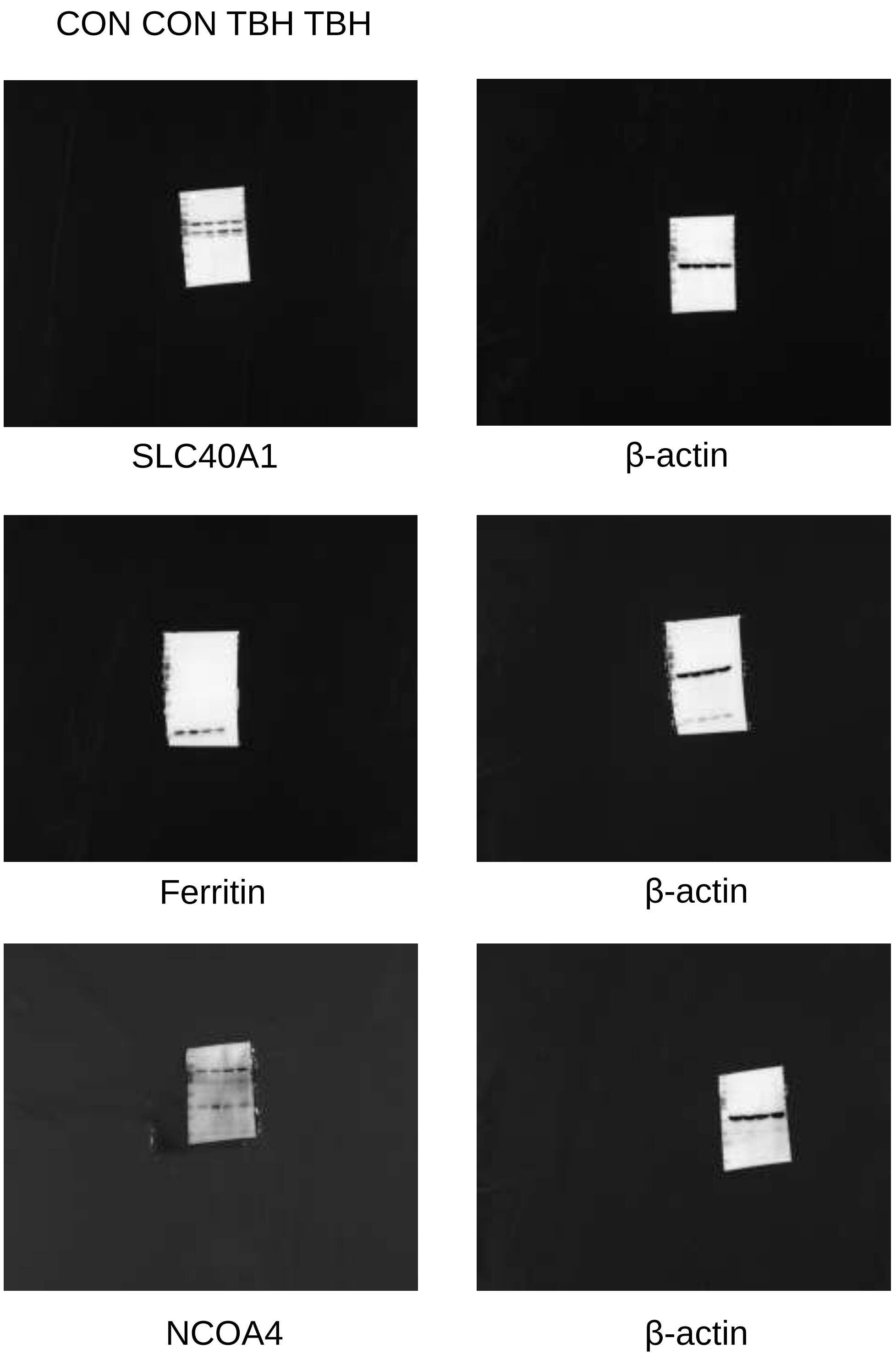

CON CON TBH TBH
SLC40A1
β-actin
Ferritin
β-actin
NCOA4
β-actin

Figure S3A

CON NC siTFRC-1 siTFRC-2 siTFRC-3

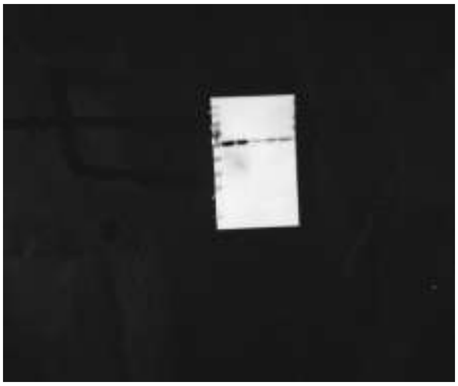

TFRC

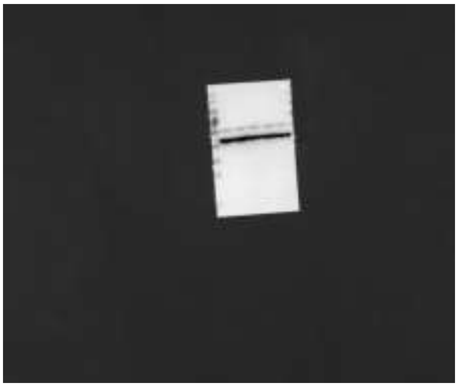

β-actin

CON NC siNCOA4-1 siNCOA4-2 siNCOA4-3

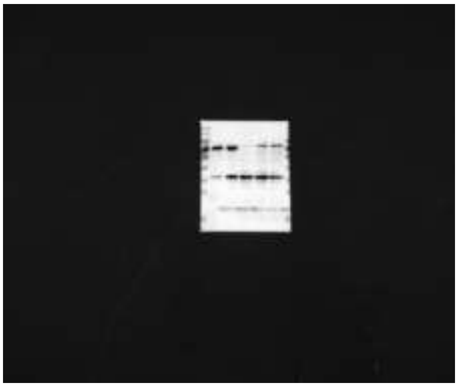

NCOA4

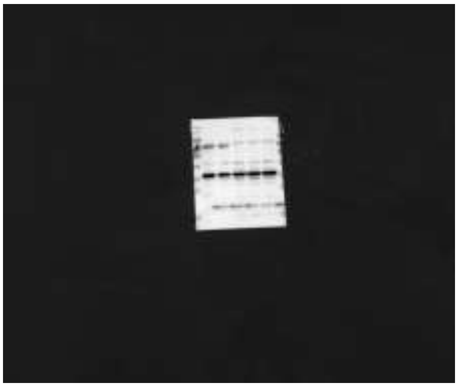

β-actin

Figure 2E

CON TBH NC siNCOA4 siTFCR siSLC40A1

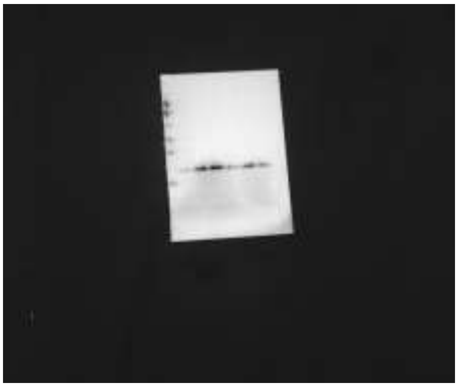

P21

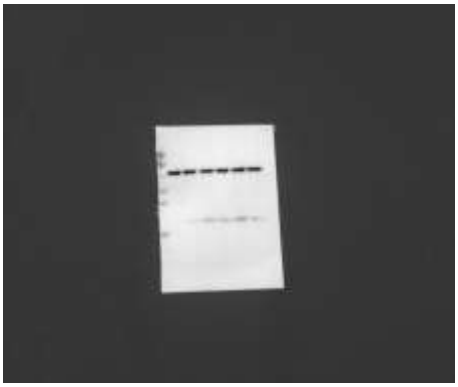

β-actin

CON TBH NC siNCOA4 siTFCR siSLC40A1

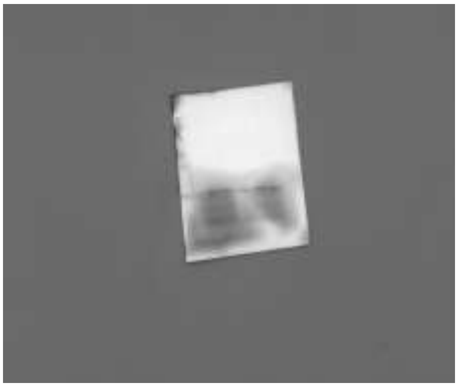

p-H2AX

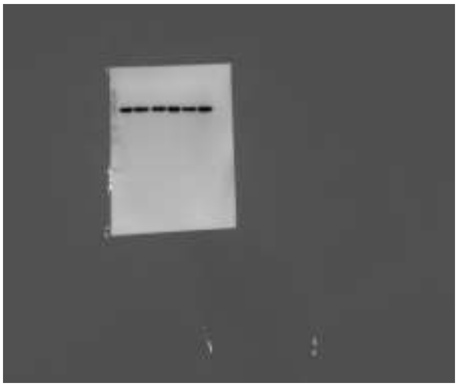

β-actin

CON TBH NC siNCOA4 siTFCR siSLC40A1

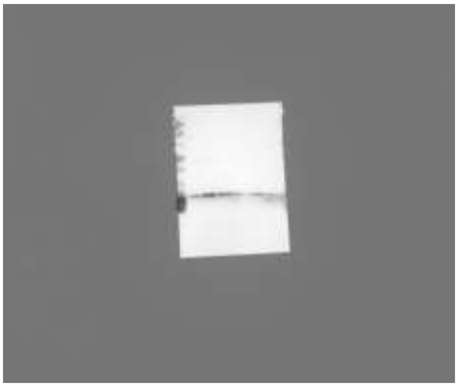

IL-6

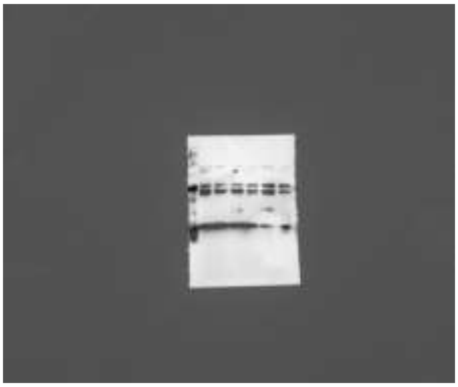

β-actin

Figure 3A

CON TBH(6h) TBH(12h) TBH(24h) TBH(48h)

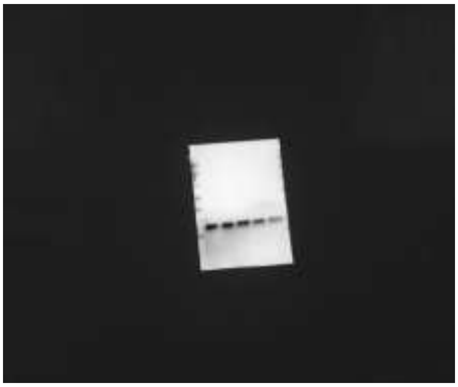

Ferritin

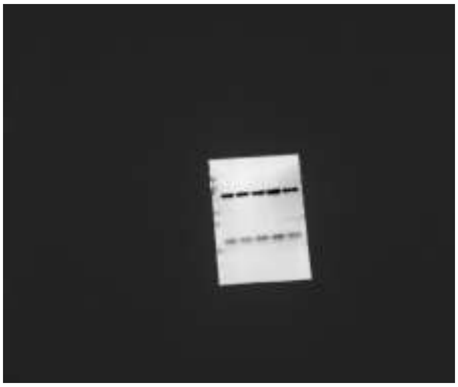

β-actin

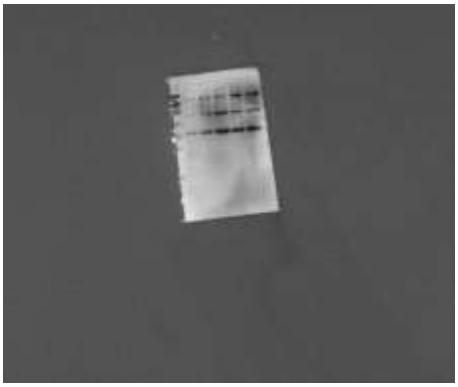

NCOA4

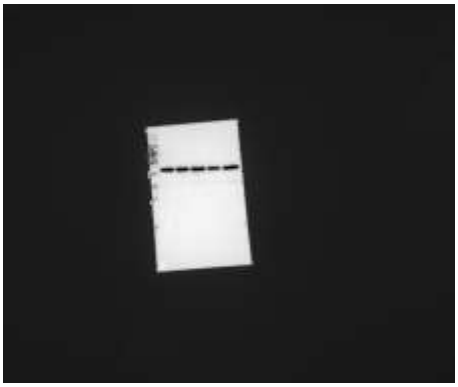

β-actin

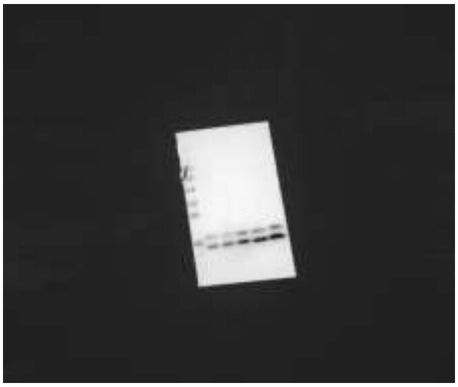

LC3B

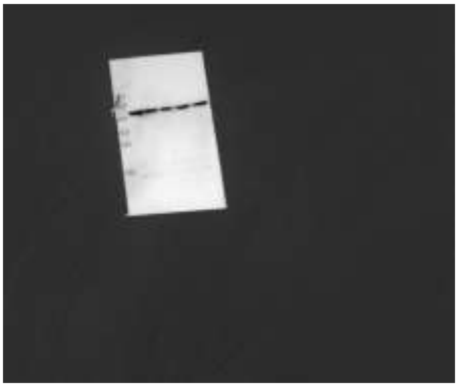

β-actin

Figure 3C

CON TBH NC siNCOA4

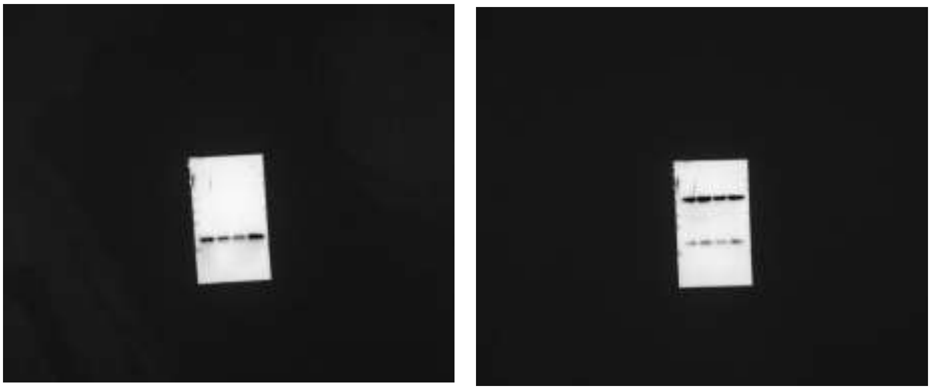

Ferritin   β-actin

Figure 3F

CON NC Ferritin(+)

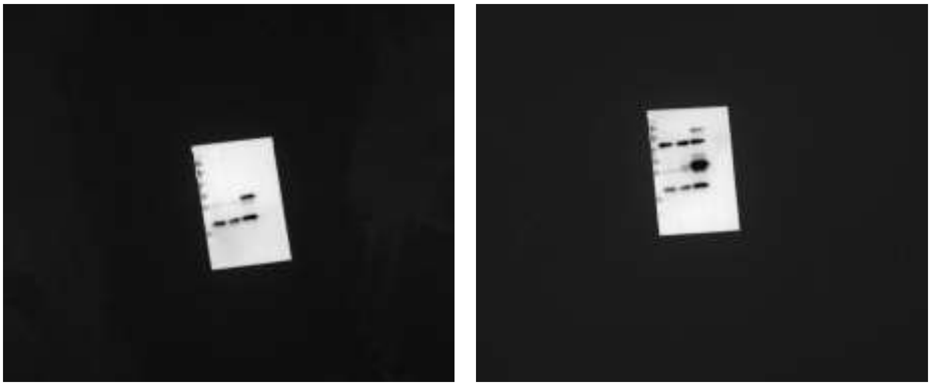

Ferritin   β-actin

Figure 4B

CON CON FAC FAC FAC+DFO FAC+DFO

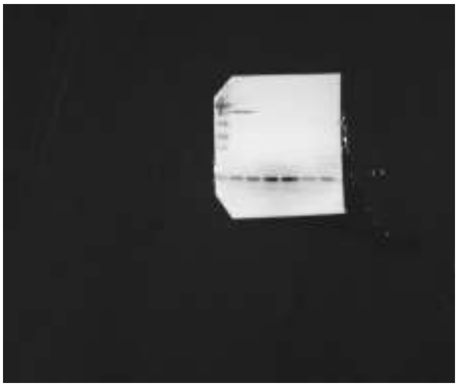

p-H2AX

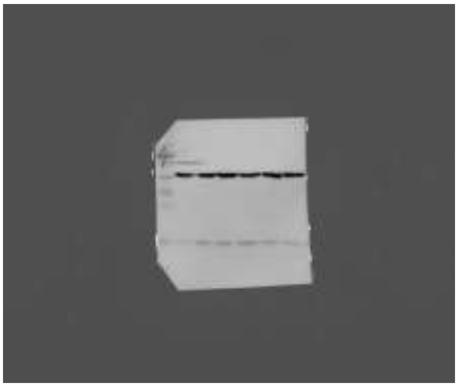

β-actin

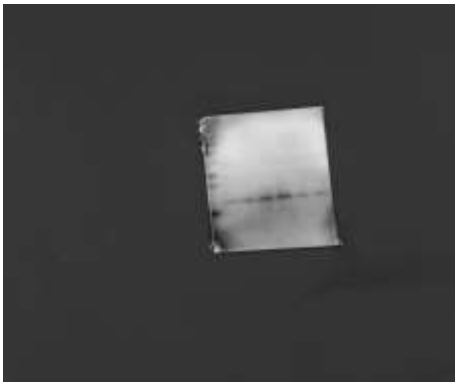

P21

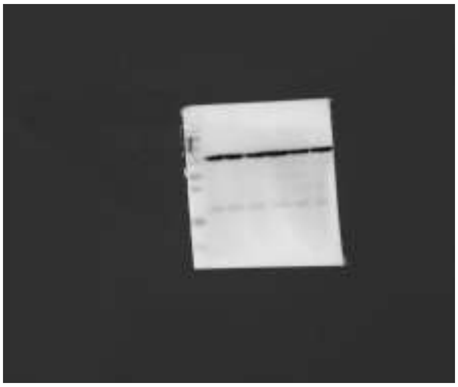

β-actin

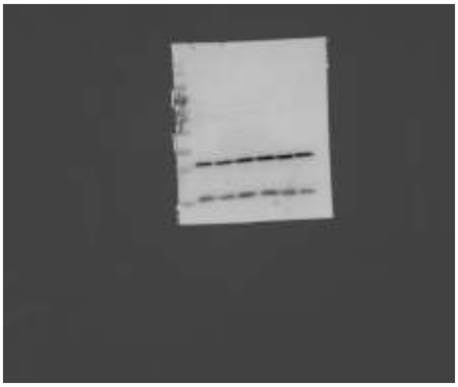

IL-6

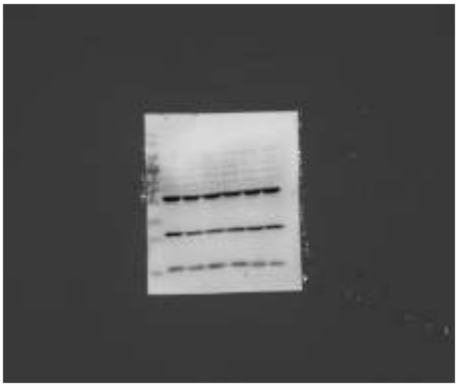

β-actin

Figure 4E

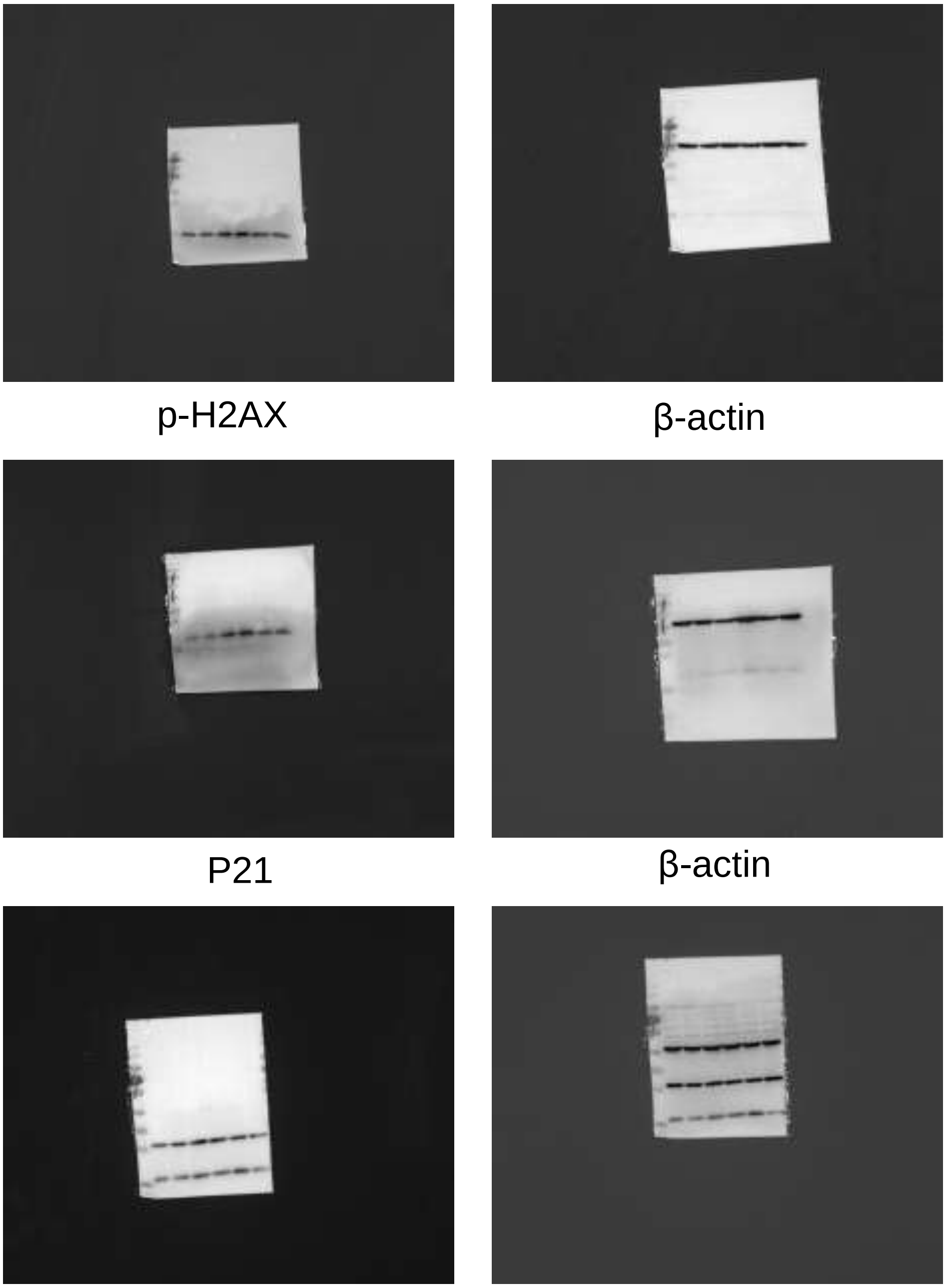
CON CON TBH TBH TBH+DFO TBH+DFO
p-H2AX
β-actin
P21
β-actin
IL-6
β-actin

Figure 4J

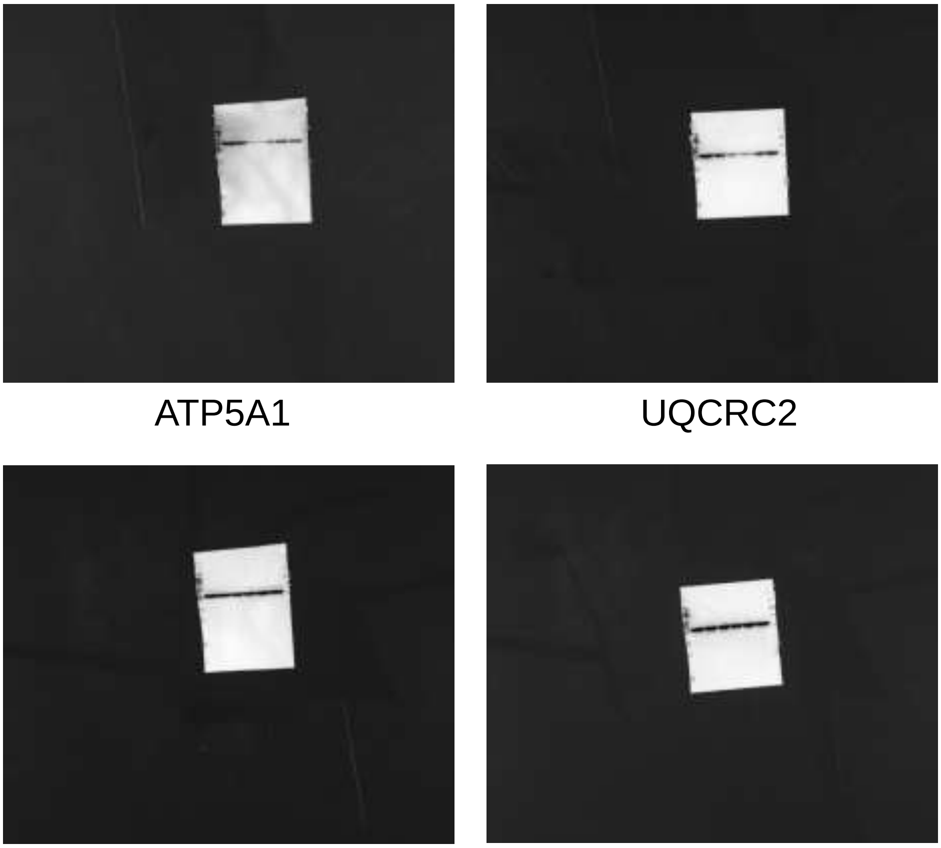

Figure 4J

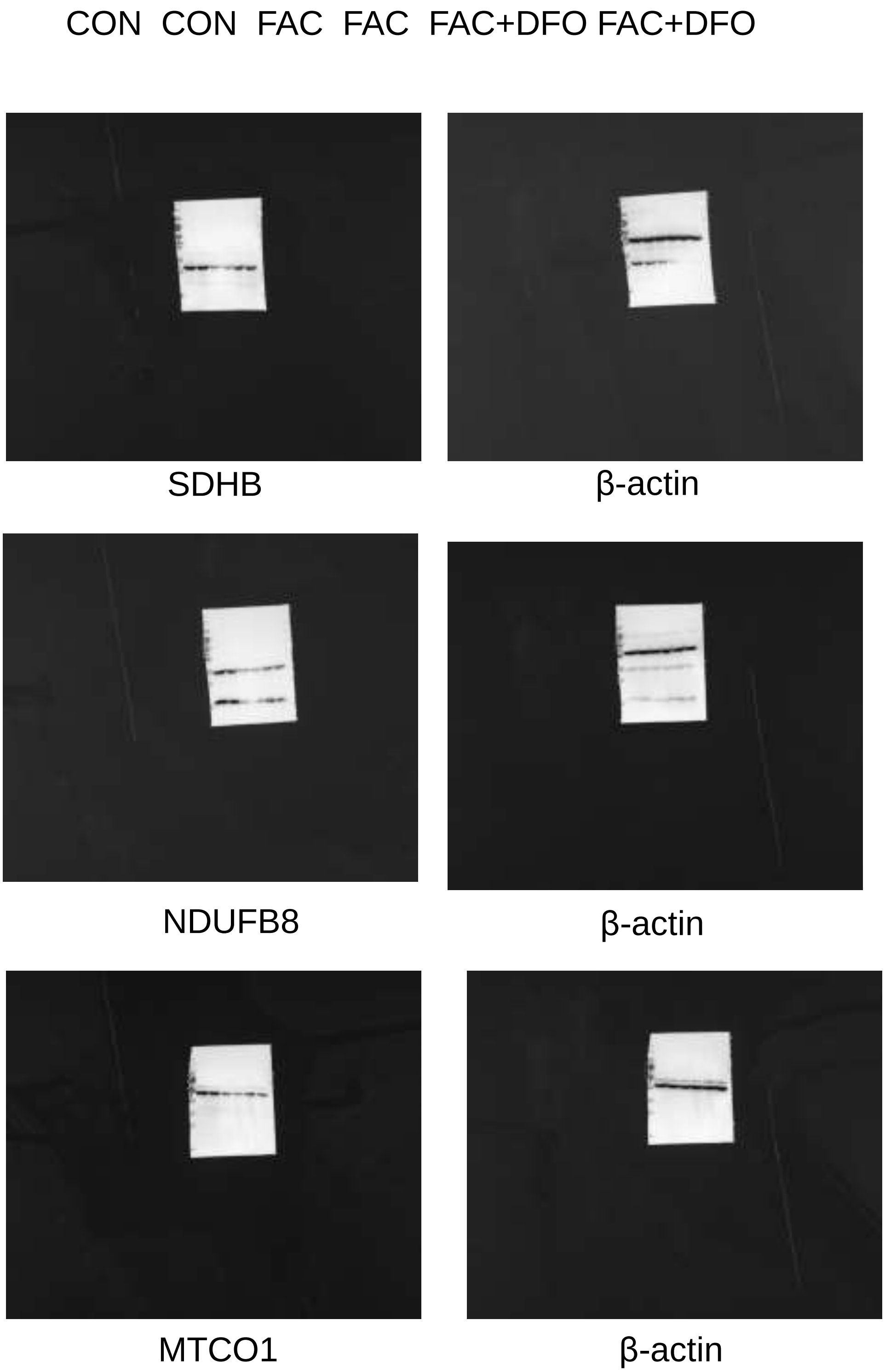
CON CON FAC FAC FAC+DFO FAC+DFO
SDHB
β-actin
NDUFB8
β-actin
MTCO1
β-actin

Figure 4L

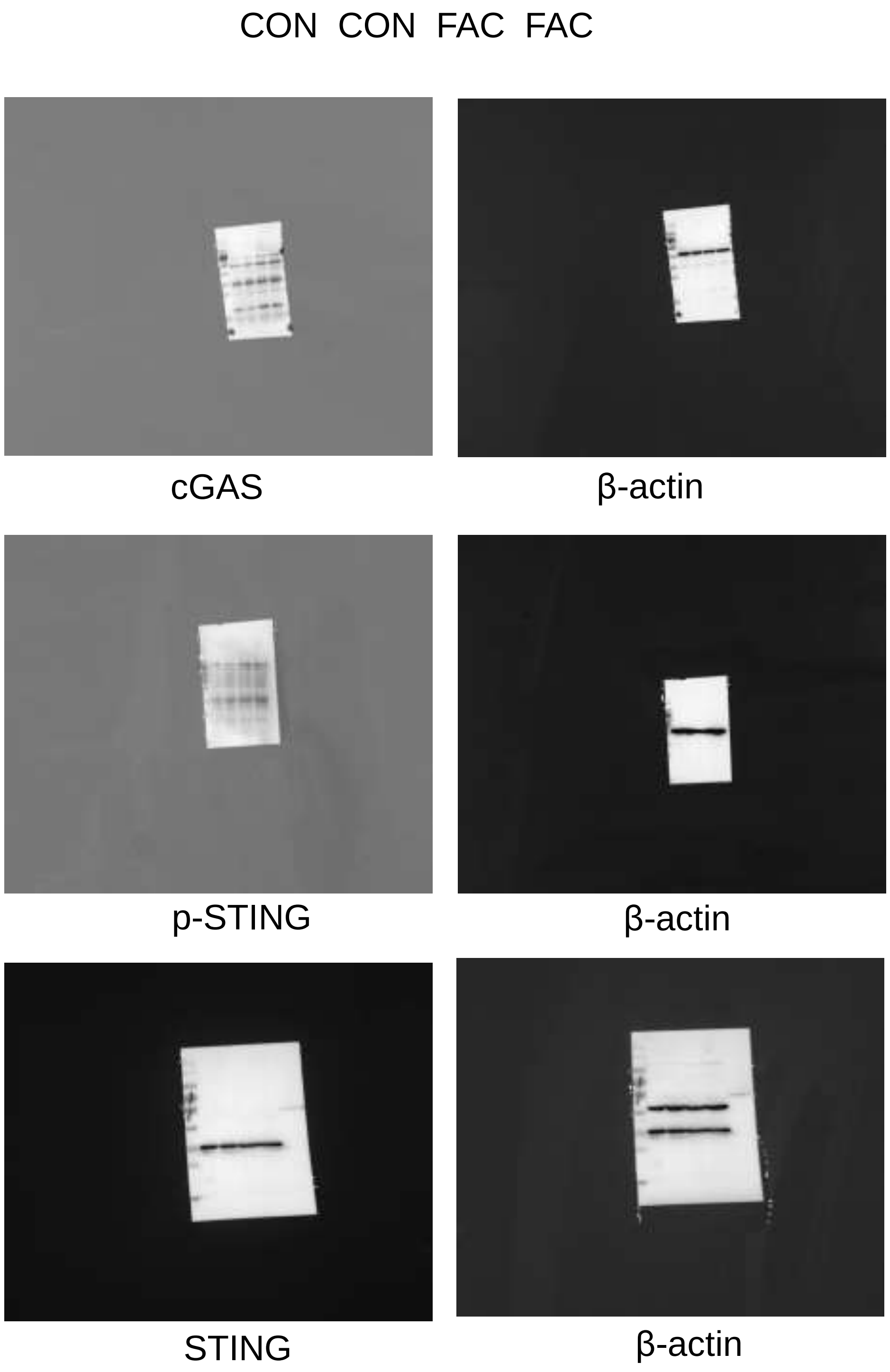

Figure 4L

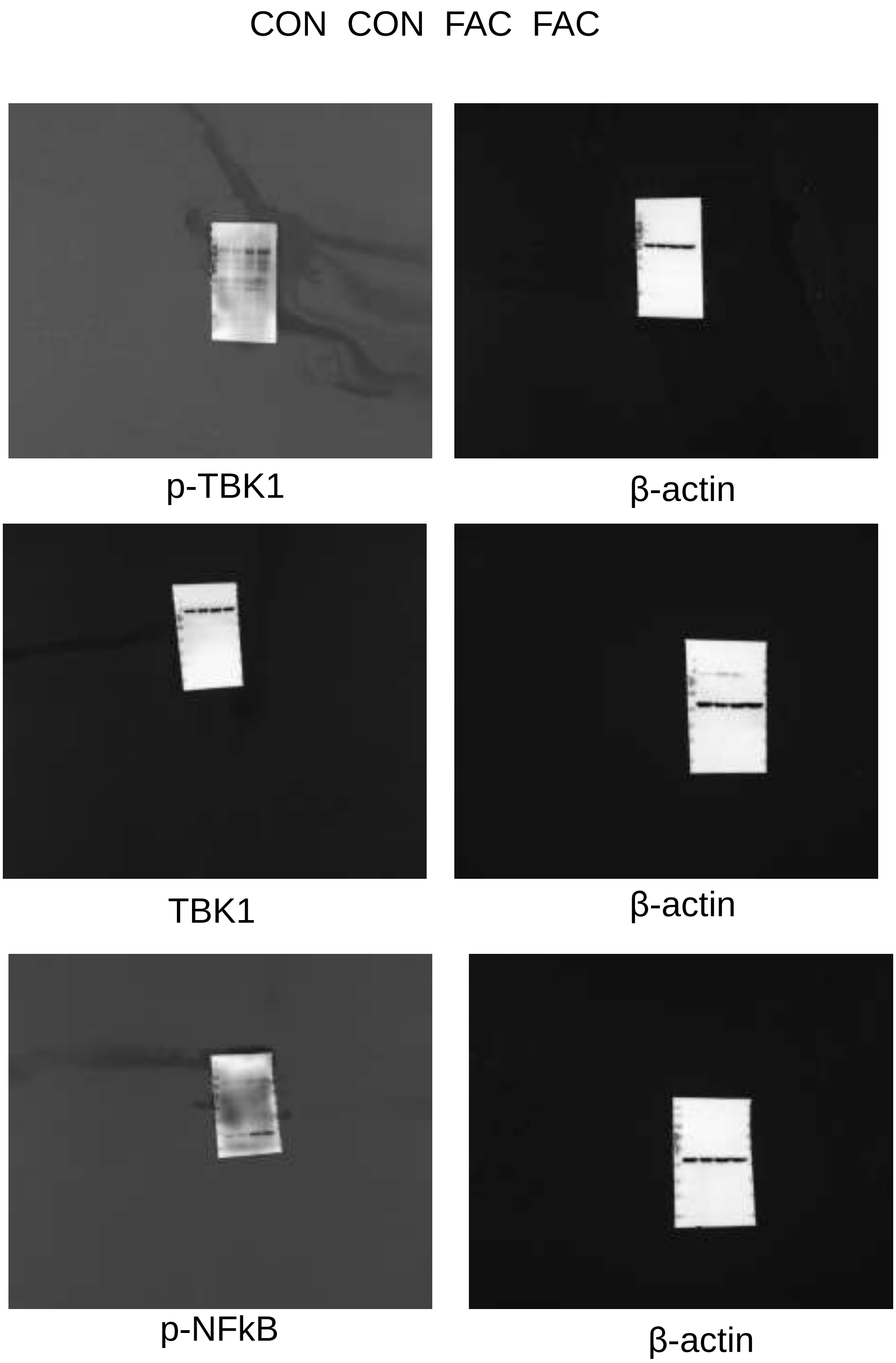

Figure 4L

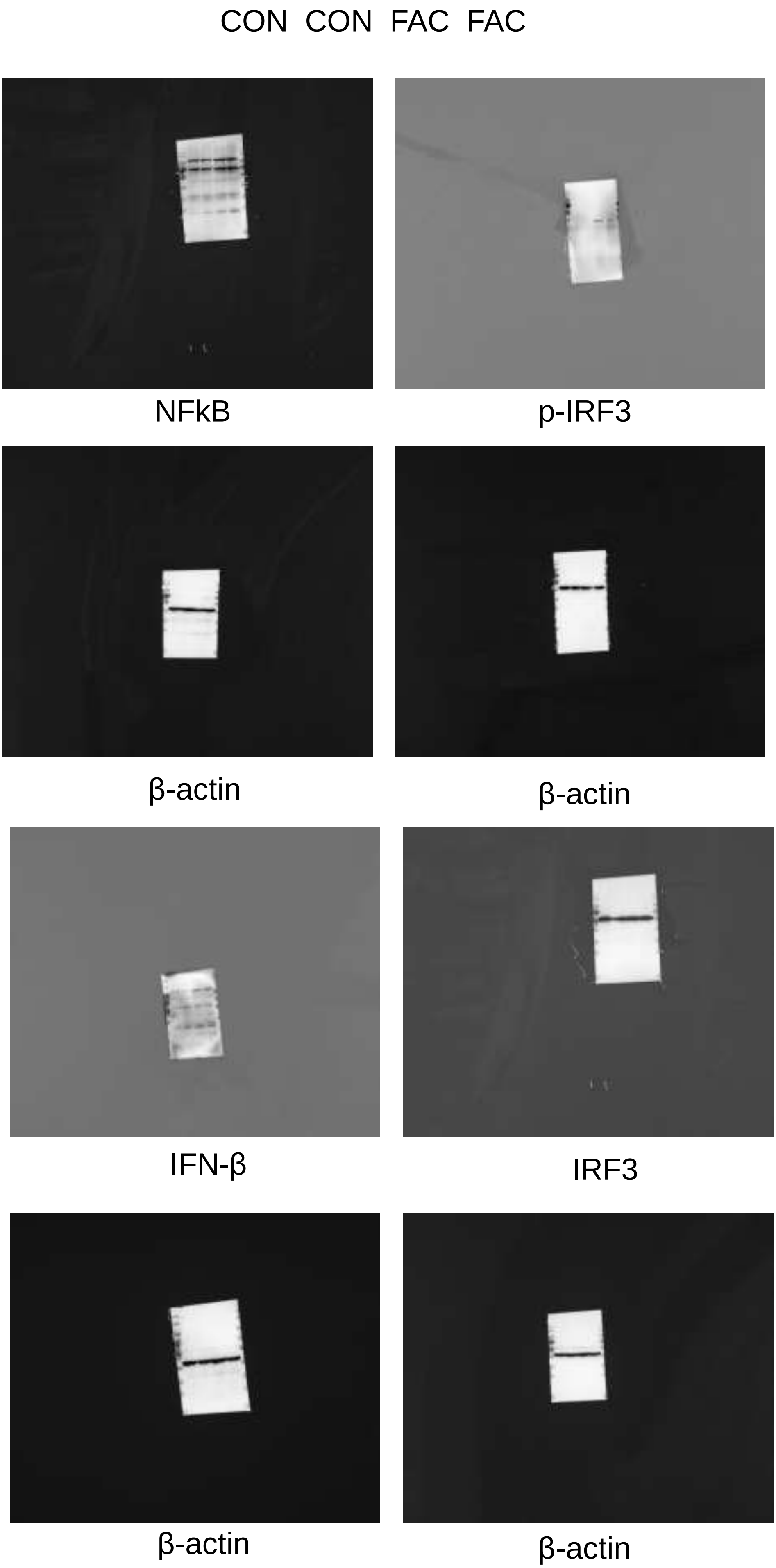
CON CON FAC FAC
NFkB
p-IRF3
β-actin
β-actin
IFN-β
IRF3
β-actin
β-actin

Figure 5I

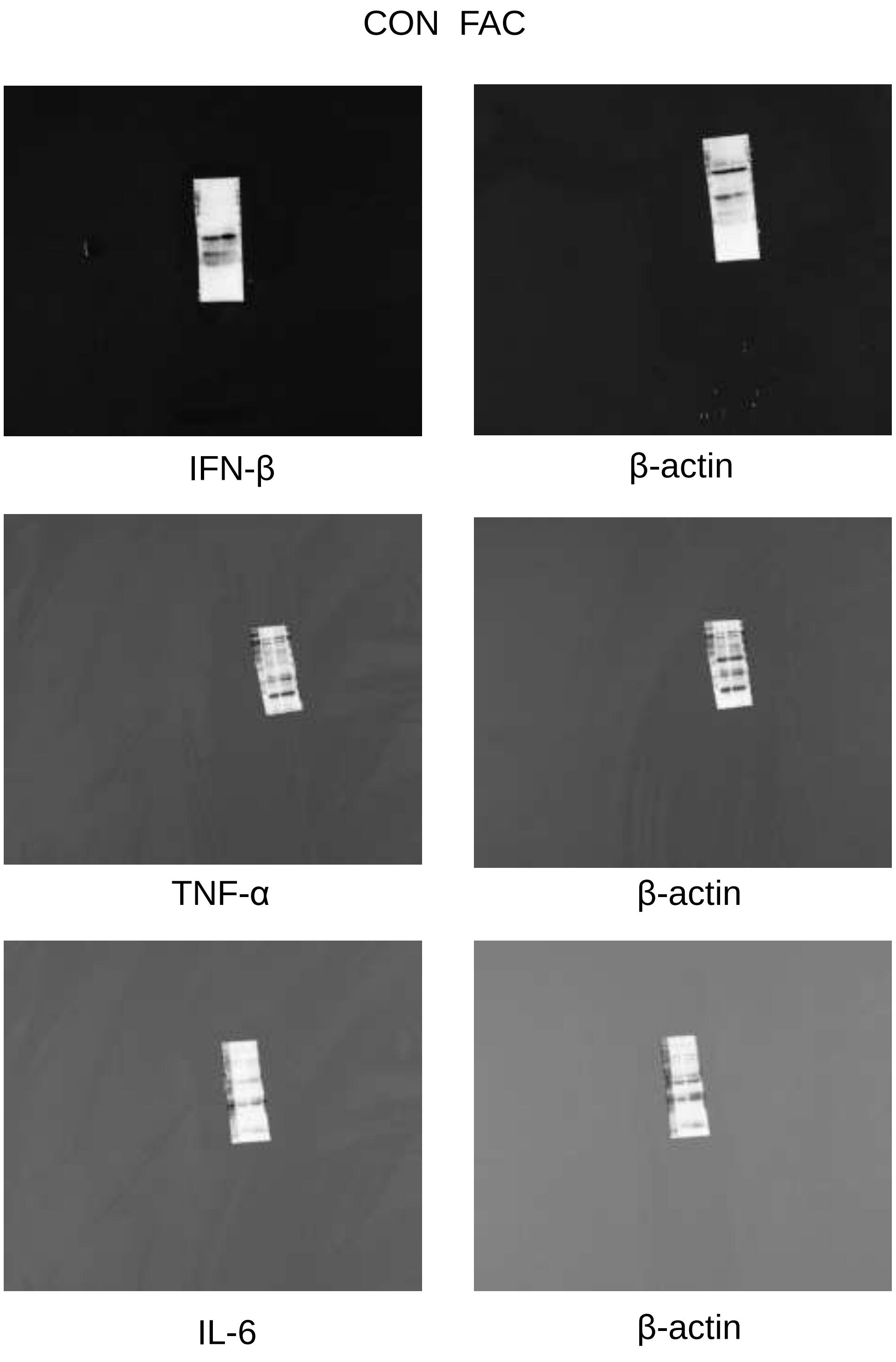

Figure 5J

CON FAC

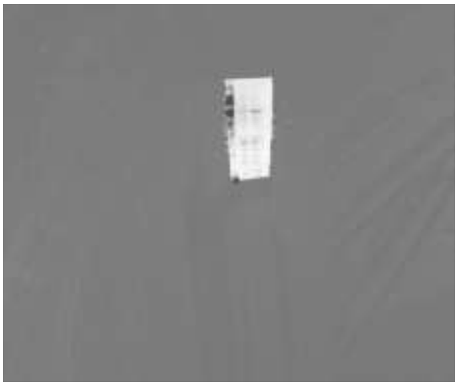

cGAS

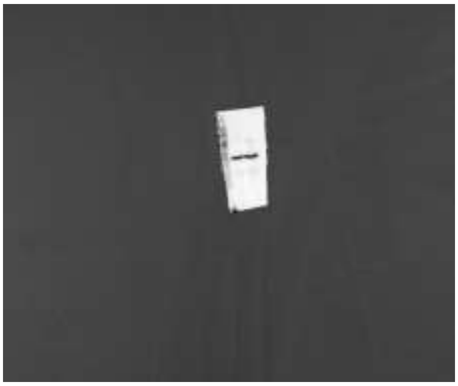

β-actin

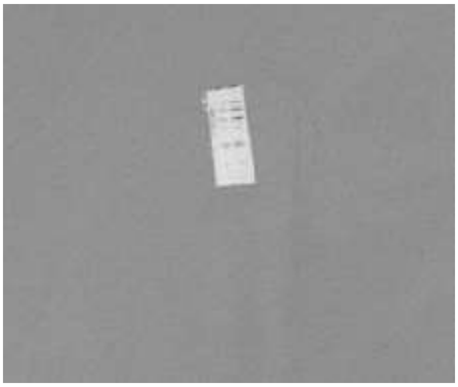

p-STING

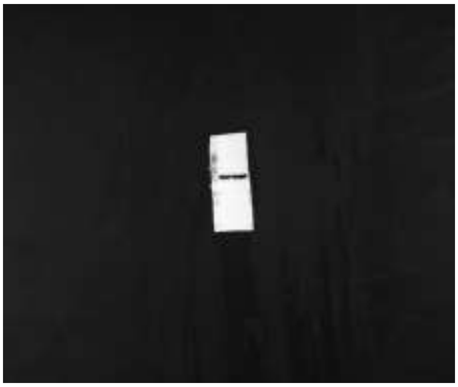

β-actin

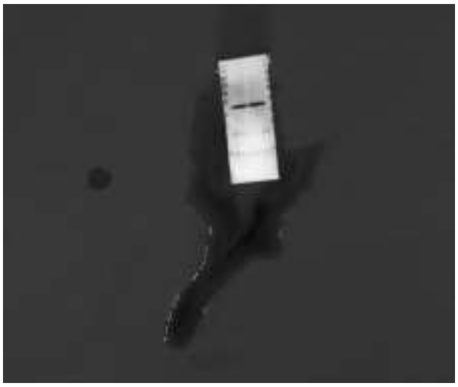

STING

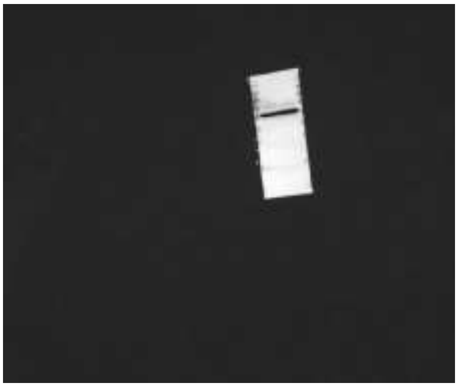

β-actin

Figure 6H

CON D-gal

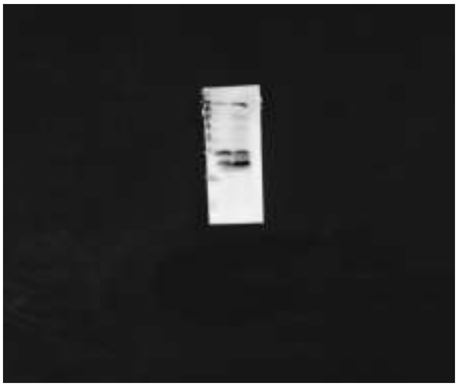

NCOA4

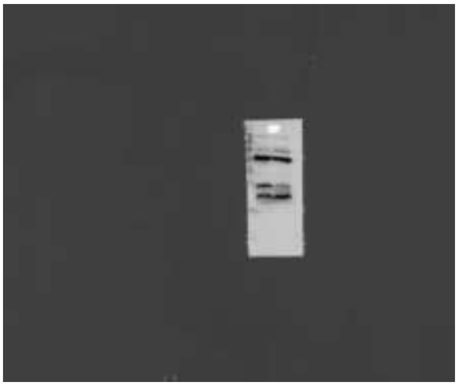

β-actin

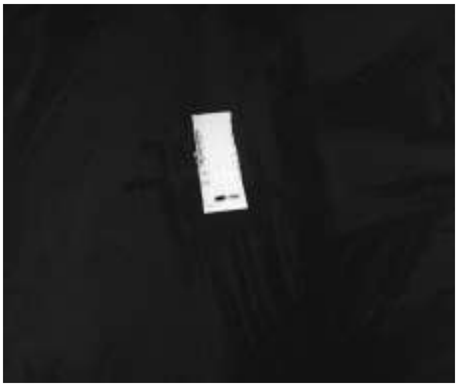

Ferritin

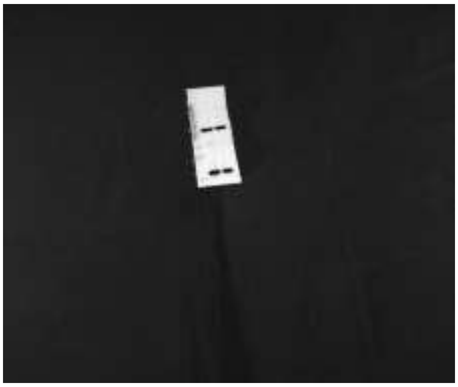

β-actin

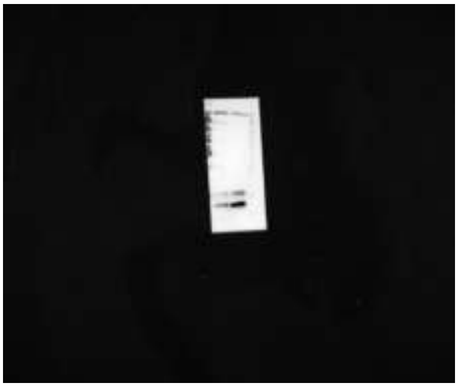

LC3B

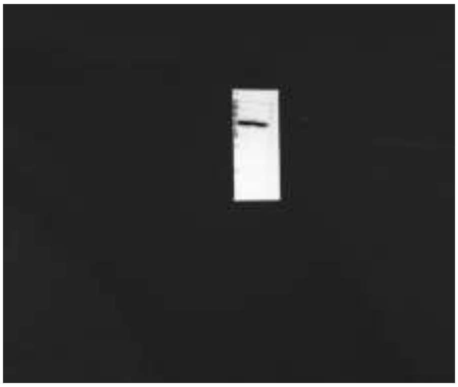

β-actin

Figure 6H

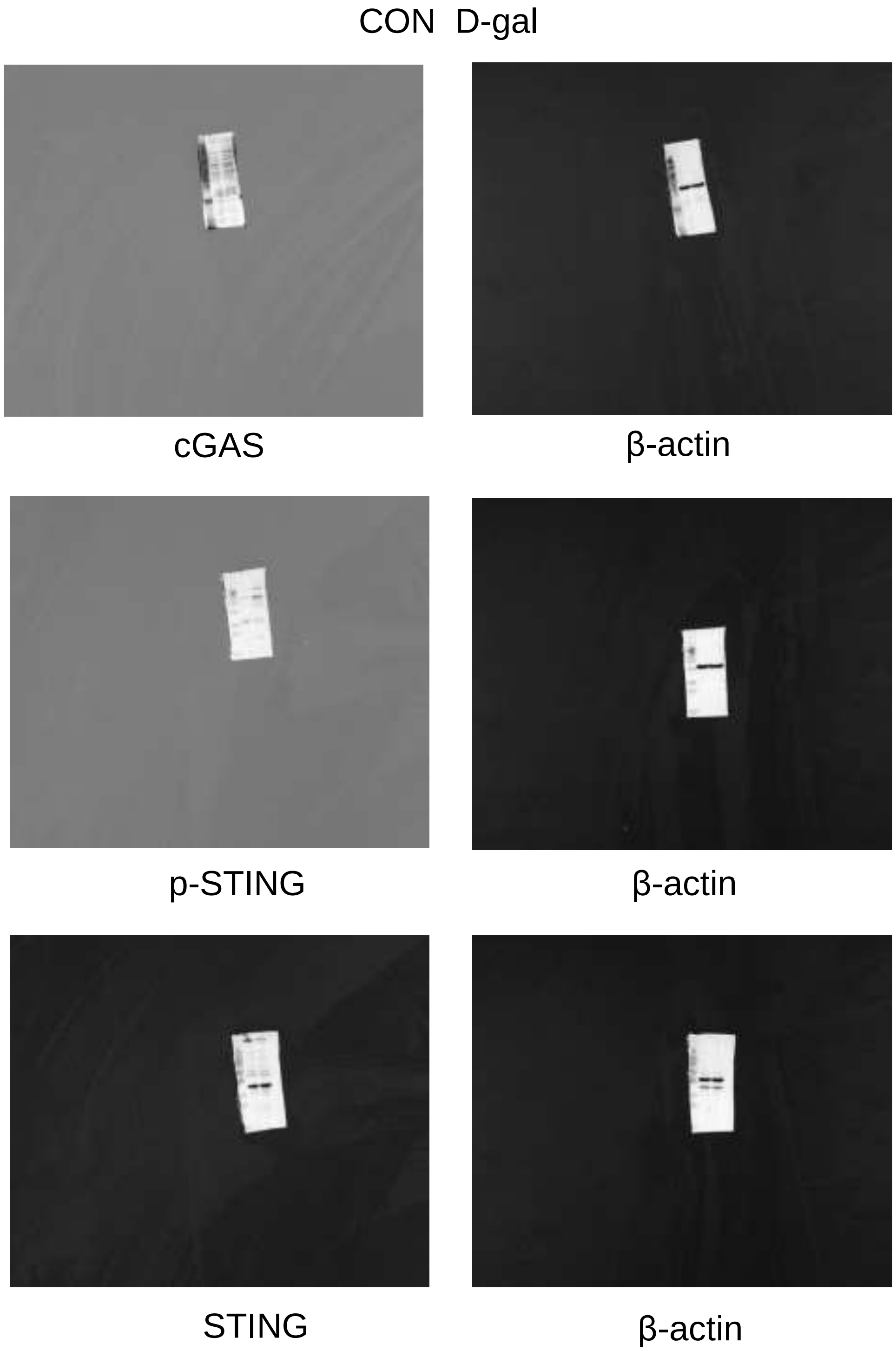

Figure S6A

CON CON TBH TBH TBH+Mito-T TBH+Mito-T

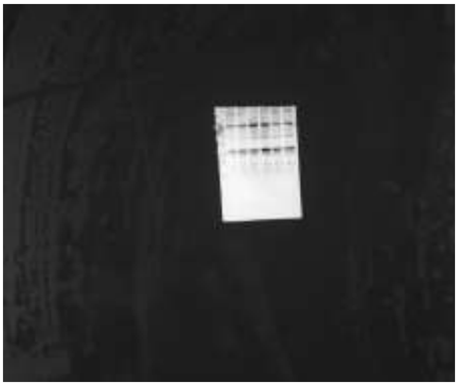

NCOA4

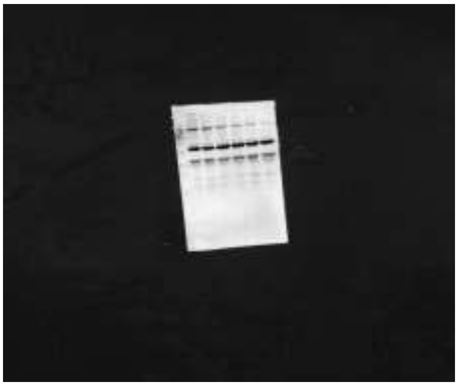

β-actin

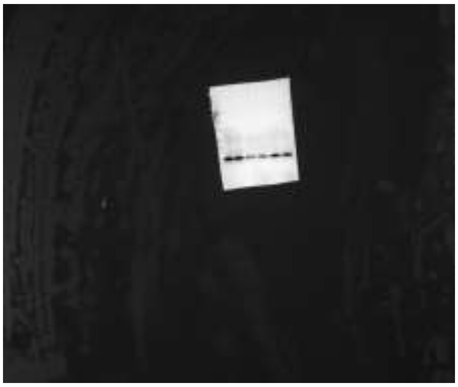

Ferritin

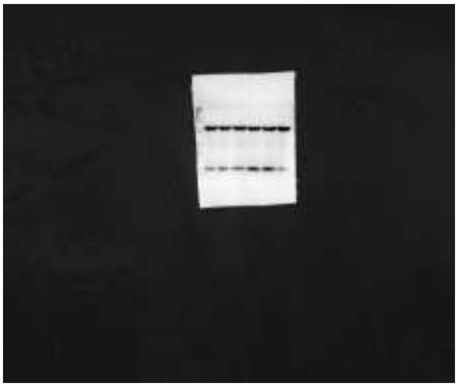

β-actin
